# Longitudinal single-cell analysis of glucagon-like peptide-2 treatment in patients with short bowel syndrome

**DOI:** 10.1172/jci.insight.194497

**Published:** 2025-08-07

**Authors:** Yumi Kudo, Kentaro Miyamoto, Shohei Suzuki, Akihiko Chida, Anna Tojo, Mai Hasegawa, Arina Shigehara, Ikuko Koya, Yoshinari Ando, Masayasu Sato, Aya Kondo, Tomoko Kumagai, Harunori Deguchi, Yoshiki Sugiyama, Yoko Ito, Koji Shirosaki, Satoko Yamagishi, Yutaro Maeda, Hiroki Kanamori, Motohiro Kano, Mototoshi Kato, Hanako Tsujikawa, Yusuke Yoshimatsu, Kaoru Takabayashi, Koji Okabayashi, Takanori Kanai, Naoki Hosoe, Motohiko Kato, Jonathan Moody, Chung-Chau Hon, Tatsuo Kuroda, Yohei Yamada, Akihiro Fujino, Tomohisa Sujino

**Affiliations:** 1 Department of Pediatric Surgery, and; 2 Division of Gastroenterology and Hepatology, Department of Internal Medicine, Keio University School of Medicine, Shinanomachi, Shinjuku-ku, Tokyo, Japan.; 3 Research Department, R&D Division, Miyarisan Pharmaceutical Co., Ltd., Saitama, Japan.; 4 Center for Diagnostic and Therapeutic Endoscopy, Keio University School of Medicine, Shinanomachi, Shinjuku-ku, Tokyo, Japan.; 5 Laboratory for Regulatory Genomics, RIKEN Center for Integrative Medical Sciences, Yokohama, Kanagawa, Japan.; 6 Department of Pediatric Surgery, Tokyo Metropolitan Children’s Medical Center, Fuchu-shi, Tokyo, Japan.; 7 Department of Pathology, Keio University School of Medicine, Shinanomachi, Shinjuku-ku, Tokyo, Japan.; 8 Department of Diagnostic Pathology, National Hospital Organization Saitama Hospital, Wako-shi, Saitama, Japan.; 9 Department of Surgery, Keio University School of Medicine, Shinanomachi, Shinjuku-ku, Tokyo, Japan.; 10 Kanagawa Children’s Medical Center, Yokohama, Kanagawa, Japan.; 11 Department of Multidimensional Analysis of Gastrointestinal Biology, Sakaguchi Laboratory, Keio University School of Medicine, Shinanomachi, Shinjuku-ku, Tokyo, Japan.; 12 Keio Global Research Institute, Keio University, Mita, Minato-ku, Tokyo, Japan.

**Keywords:** Clinical Research, Gastroenterology, Drug therapy

## Abstract

**BACKGROUND:**

Glucagon-like peptide-2 (GLP-2) analogs are used clinically to enhance nutrient absorption in patients with short bowel syndrome (SBS); however, the precise mechanism remains unclear. To address this, the study aimed to clarify the dynamics of intestinal epithelial cells and immune cells in patients with SBS treated with GLP-2 analogs.

**METHODS:**

Five male patients diagnosed with SBS, all of whom received treatment with the GLP-2 analog teduglutide, were included in the study. We conducted longitudinal single-cell RNA sequencing (scRNA-Seq) analysis of intestinal tissue from patients with SBS over a year, integrating microbiome composition analysis.

**RESULTS:**

After treatment, the α-diversity of the gut microbiome increased, indicating a more varied microbial environment. ScRNA-Seq analysis revealed a reduction of T helper 2 cells and an increase in regulatory T cells, suggesting a shift toward an immunoregulatory intestinal environment. Additionally, nutrient-absorbing enterocyte-Top2 and middle clusters expanded, enhancing the absorption capacity, whereas major histocompatibility complex class I/II–expressing enterocyte-Top1 cells declined, potentially modulating immune responses.

**CONCLUSION:**

The study findings indicate that GLP-2 analogs reshape intestinal immunity and microbiota, fostering a less inflammatory environment while promoting nutrient uptake efficiency. These insights offer a deeper understanding of the role of GLP-2 analogs in gut adaptation and provide a foundation for refining clinical strategies for SBS treatment.

**FUNDING:**

This work was supported by Sakaguchi Memorial Foundation, Grants-in-Aid from the Japanese Society for the Promotion of Science (JSPS) (21K18272, 23H03665, 23H02899, 23K27590, 25K22627, 23K08037), JST FOREST(21457195), and the Takeda Japan Medical Office Funded Research Grant 2022.

## Abstract 2

GLP-2 analogs induce nutrient-absorbing enterocyte clusters and immune suppressive cells with icreased alpha-diversity of microbiota in small intestine.

## Graphical abstract

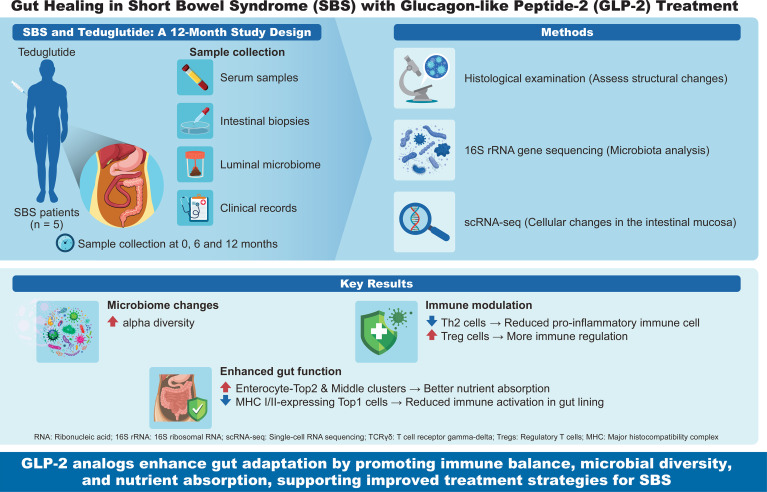

## Introduction

Glucagon-like peptide-2 (GLP-2) is an intestinal hormone crucial for maintaining gut health and function. It is derived from the pro-glucagon gene, which produces multiple peptides, including GLP-1 and GLP-2, primarily secreted by intestinal L cells (1). GLP-2 exerts its effects by binding to the GLP-2 receptor in subepithelial myofibroblasts and stimulating the expression and secretion of insulin-like growth factor-1 (IGF-1) (2). Increased levels of IGF-1 and IGF-2 are associated with gut growth, particularly in the mucosal and muscularis regions. IGF-1, in turn, activates IGF-1 receptor (IGF1R) in the intestinal epithelial cells, thereby promoting proliferation (3–6). GLP-2 stimulates intestinal epithelial growth and proliferation by activating intracellular signaling pathways, such as protein kinase A, protein kinase B, and extracellular signal–regulated kinase 1/2, thereby enhancing cell survival, proliferation, and nutrient absorption (4, 6–11). These pathways contribute to increased villus height, reduced apoptosis, and enhanced proliferation of crypt cells.

GLP-2 plays multiple roles in digestive function: (a) It promotes intestinal mucosal growth by stimulating cell proliferation and inhibiting apoptosis, thereby increasing the surface area for nutrient absorption. (b) It maintains intestinal barrier integrity and prevents harmful bacterial invasion. (c) It prolongs intestinal transit time, thereby optimizing nutrient digestion and absorption (12–15). Recently, GLP-2 analogs have been clinically used for the treatment of short bowel syndrome (SBS) (16–21). However, the cellular and microbiome-level effects of GLP-2 analogs remain unclear. To address this knowledge gap, we conducted a longitudinal single-cell RNA-sequencing (scRNA-Seq) and 16S ribosomal RNA (16S rRNA) sequencing study to investigate how GLP-2 analogs influence intestinal cell populations and microbiome compositions over time. By employing these advanced techniques, we aimed to characterize the immunological and microbiological changes associated with GLP-2 treatment, providing deeper insights into its role in gut adaptation and its potential clinical implications for SBS management.

## Results

### GLP-2 analog therapy is effective for patients with SBS.

**Figure 1:**
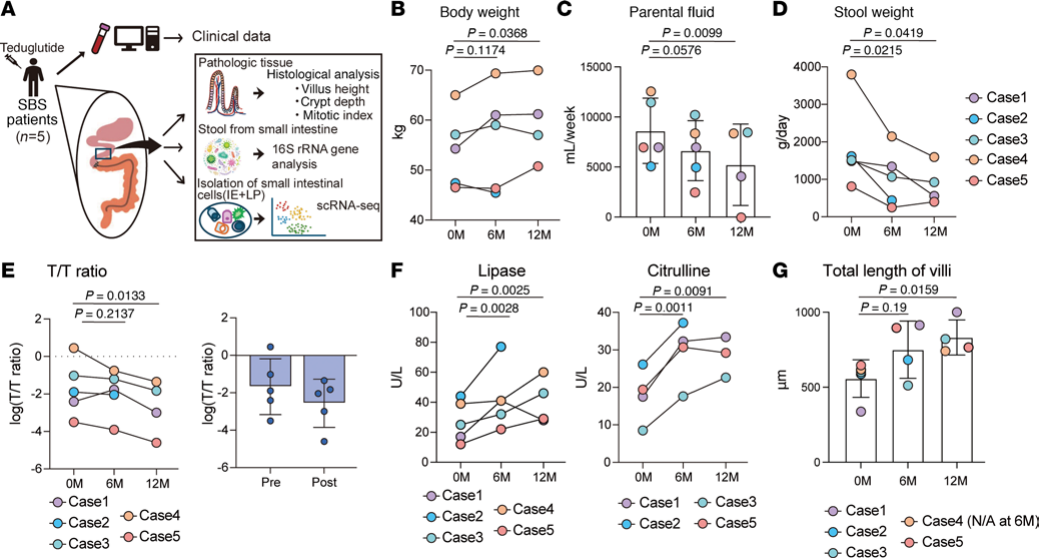
GLP-2 analog is effective for patients with SBS. (**A**) Study design: Five male patients with short bowel syndrome (SBS) were enrolled and treated with the glucagon-like peptide-2 (GLP-2) analog teduglutide. Serum samples, intestinal tissue biopsies, luminal microbiome samples, and clinical records were collected at 3 time points: before treatment (0 months, 0M), 6 months after initiation (6M), and 12 months after initiation (12M). Intestinal microbiomes were analyzed using 16S ribosomal RNA (16S rRNA) sequencing, and intestinal tissue samples underwent histological examination and single-cell RNA sequencing (scRNA-Seq). (**B** and **D**) Body weight (**B**) and stool output (**D**): Each line represents changes in body weight or stool weight for an individual patient across the 3 time points. (**C**) Parenteral fluid volume: Each dot represents the volume of parenteral fluid administered to an individual patient across the 3 time points; bars indicate the mean ± SD. (**E**) The eicosatrienoic acid/arachidonic acid (T/T) ratio in blood: The left panel shows changes in the T/T ratio at the 3 time points. The right panel compares the T/T ratio before treatment (0M) with posttreatment (either 6M or 12M, with the later time point selected). Each dot represents an individual sample; bars indicate the mean ± SD. Values are log-transformed. (**F**) Blood biomarkers: Changes in blood levels of lipase and citrulline at the 3 time points. (**G**) Intestinal villi length: Changes in the total villus length (sum of villus height and crypt depth) at the 3 time points based on histological analysis. Each dot represents an individual sample; bars indicate the mean ± SD. Due to missing crypt depth data for case 4 at 6M, this data point was excluded. Statistical analyses included the 1-tailed paired *t* tests (**B**–**F**) and the Mann-Whitney test (**G**). IE, intestinal epithelium; LP, lamina propria; N/A, not available due to missing crypt depth data.

This study enrolled 5 male patients diagnosed with SBS who were treated with the GLP-2 analog teduglutide (Supplemental Table 1; supplemental material available online with this article; https://doi.org/10.1172/jci.insight.194497DS1; cases 1–5). Clinical data, serum samples, intestinal tissue biopsies, and luminal content microbiota were collected at 3 time points: baseline (prior to treatment initiation, 0 months [0M]), 6 months after treatment (6M), and 12 months after treatment (12M). Intestinal microbial composition was assessed via 16S rRNA gene sequencing, while intestinal biopsies underwent histological evaluation and scRNA-Seq (Figure 1A). All patients retained portions of the duodenum and jejunum, with complete resection of the ileum. The residual small intestinal lengths varied: case 1 had 30 cm of jejunum; case 2, 40 cm of duodenum; case 3, 35 cm of duodenum; case 4, 10 cm of jejunum; and case 5, 40 cm of jejunum. Teduglutide treatment was initiated between 6 months and 13 years following small intestinal resection. Prior to treatment, all patients required parenteral nutrition via central venous access due to inadequate oral intake.

After 12 months of teduglutide therapy, 4 of the 5 patients exhibited weight gain, with the exception of case 2 (Figure 1B). Parenteral fluid requirements decreased in all cases, and total stool volume was reduced, indicating improved intestinal function (Figure 1, C and D). Serum biochemical analysis revealed a posttreatment decline in the eicosatrienoic acid/arachidonic acid (T/T) ratio (Figure 1E). Concurrently, levels of citrulline and lipase, which are biomarkers of intestinal absorptive capacity, increased, consistent with enhanced nutrient uptake (Figure 1F). Histologic assessment demonstrated increased villus and crypt height over the 12-month period (Figure 1G; Supplemental Figure 1, A–E; and Supplemental Table 2), supporting the role of GLP-2 analogs in promoting mucosal adaptation. Collectively, these data suggest that teduglutide treatment fosters mucosal growth, improves nutrient absorption, and contributes to nutritional rehabilitation in patients with SBS.

### GLP-2 analog therapy enhances microbial diversity and functional capacity without uniform taxonomic shifts in SBS.

**Figure 2:**
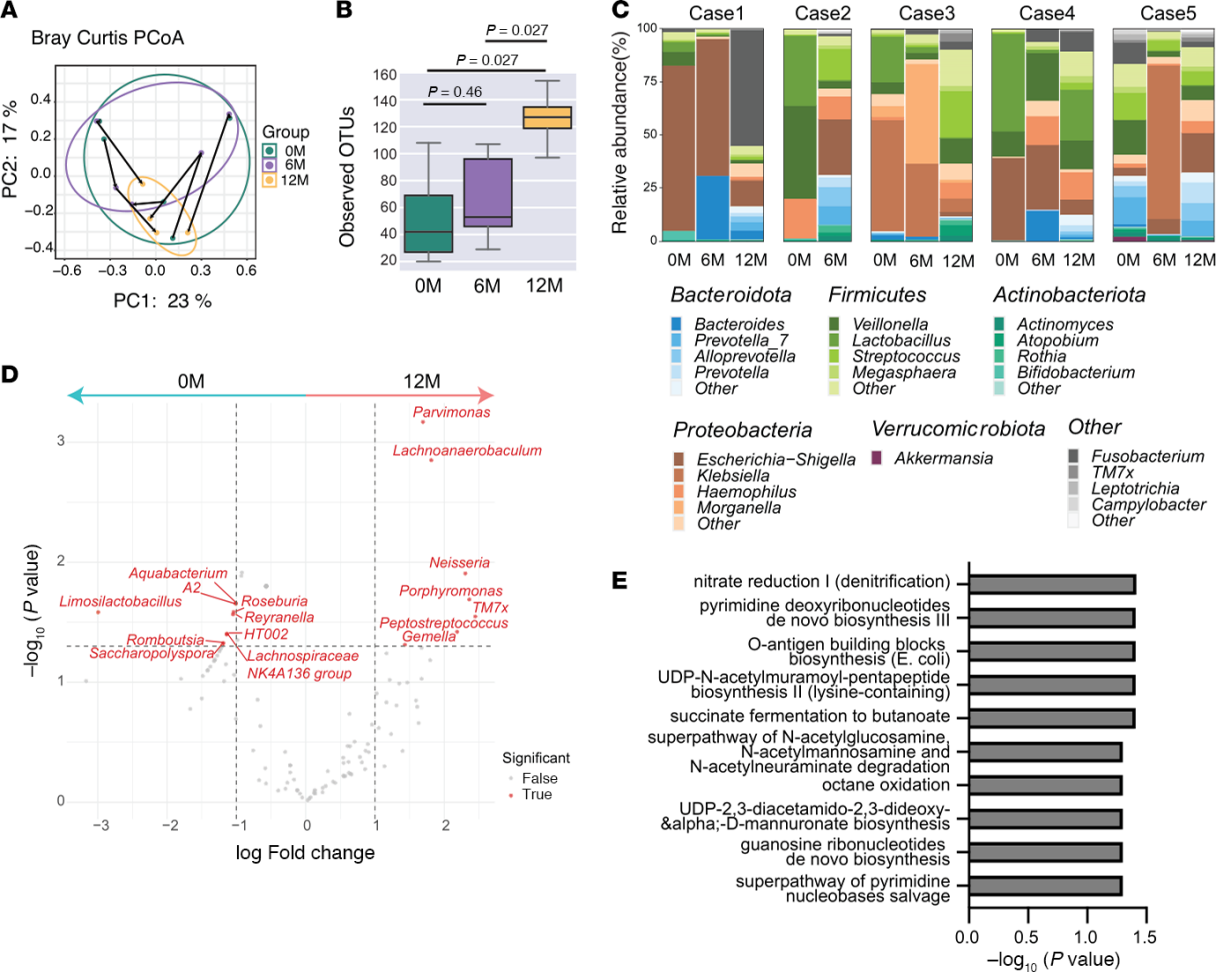
GLP-2 analog therapy enhances microbial diversity and functional capacity without uniform taxonomic shifts in SBS. (**A**) β-diversity analysis: Principal coordinate analysis (PCoA) based on the Bray-Curtis dissimilarity index to assess gut microbiota differences among samples. (**B**) α-diversity analysis: Comparison of microbial diversity at 0 months (0M), 6M, and 12M based on observed operational taxonomic units (OTUs). The box shows the interquartile range (IQR), with the line inside indicating the median. The whiskers extend to the smallest and largest values within 1.5 times the IQR. Each dot represents an individual sample. (**C**) Microbial composition: Relative abundance of gut microbiota in each sample, represented by bacterial genus and categorized by phylum. (**D**) Differentially abundant taxa: Log2 fold change (LFC) of differentially abundant genera identified through Analysis of Compositions of Microbiomes with Bias Correction 2 (AMCOM-BC2). Genera with LFC > 1 were positively enriched, while those with LFC < –1 were negatively enriched. (**E**) MetaCyc functional pathways: Predicted MetaCyc functional pathways that showed significant temporal differences by Friedman test and exhibited increased abundance at 12M compared with 0M and/or 6M are shown. The *x*-axis represents the –Log10 (*P* value) from statistical comparisons of pathway abundances. Statistical analyses included permutational multivariate 2-way ANOVA (**A**), the Mann-Whitney test (**B**), and Friedman test (**E**).

We analyzed the composition of the intestinal microbiota at 0M, 6M, and 12M using 16S rRNA gene sequencing. The gut microbiota exhibited dynamic alterations throughout the treatment period. Principal coordinate analysis based on Bray-Curtis dissimilarity and weighted and unweighted UniFrac plot revealed marked interindividual variation, with no consistent clustering pattern across time points (Figure 2A and Supplemental Figure 2A). The α-diversity, measured by observed operational taxonomic units, was significantly elevated at 12M compared with 0M and 6M, indicating increased microbial richness following treatment (Figure 2B).

The microbiota composition patterns varied across patients. Cases 1 and 3 exhibited a dominance of Proteobacteria at 0M and 6M, which decreased at 12M. Cases 2 and 4 initially harbored Firmicutes-dominant profiles, with a posttreatment increase in Bacteroides. Case 5 demonstrated transient Proteobacteria enrichment at 6M, followed by a shift in microbial community structure at 12M (Figure 2C). Differential abundance analysis comparing 0M and 12M identified increased representation of *TM7x*, *Porphyromonas*, *Neisseria*, *Peptostreptococcus*, *Lachnoanerobaculum*, *Parvimonas*, and *Gemella* following treatment. Conversely, *Limosilactobacillus*, *Saccharopolyspora*, *Romboutsia*, *Lachnospiraceae*, *HT002*, *Roseburia*, *Reyranella*, *A2*, and *Aquabacterium* declined at 12M (Figure 2D).

To infer microbial functional potential, we performed PICRUSt2-based pathway prediction. The posttreatment (12M) microbiota tended to be enriched in several metabolic pathways, including nitrate reduction, pyrimidine deoxyribonucleotides de novo biosynthesis, *O*-antigen building blocks biosynthesis, UDP-N-acetylmuramoyl-pentapeptide biosynthesis II, octane oxidation, d-mannuronate biosynthesis, guanosine ribonucleotides de novo biosynthesis, superpathway of pyrimidine nucleobases salvage, and succinate fermentation to butanoate, collectively supporting enhanced adenosine and short-chain fatty acid (SCFA) metabolism (Figure 2E). In the succinate fermentation to butanoate pathway, the enzyme commission entries for 4-hydroxybutanoyl-CoA dehydratase, 4-hydroxybutyrate dehydrogenase, and succinate-semialdehyde dehydrogenase tended to be enriched at 12M (Supplemental Figure 2B). In summary, GLP-2 analog treatment did not promote uniform expansion of specific bacterial taxa but was associated with increased microbial diversity and enrichment of SCFA-related metabolic pathways, suggesting a shift toward a more metabolically active and potentially beneficial microbiota.

### Single-cell transcriptomics reveals epithelial expansion and mucosal remodeling during GLP-2 therapy.

To characterize cellular alterations in the small intestine during GLP-2 analog therapy, we conducted scRNA-Seq on intestinal biopsy samples collected at 0M, 6M, and 12M from cases 1, 2, 3, and 5. Case 4 was excluded because of dialysis-related fluctuations in dietary intake, which could confound quantitative assessments. Additionally, 12M samples from cases 2 and 5 were not available.

**Figure 3:**
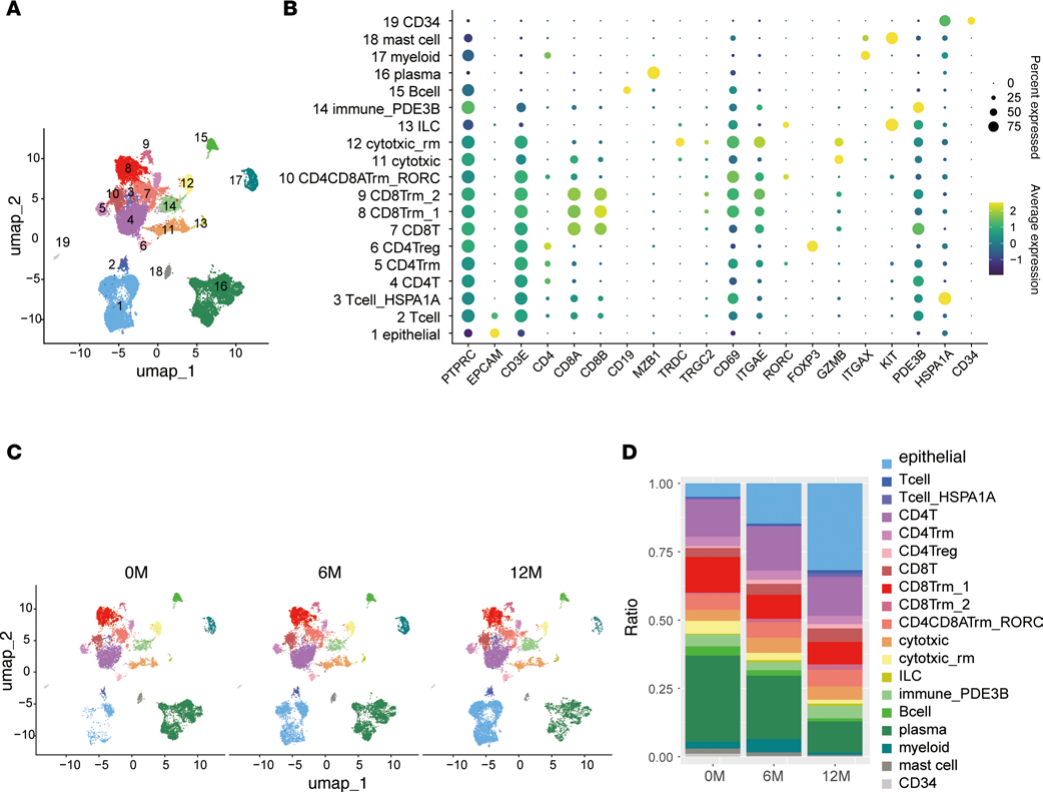
Single-cell transcriptomics reveals epithelial expansion and mucosal remodeling during GLP-2 therapy. (**A**) Uniform manifold approximation and projection (UMAP) visualization of small intestine mucosal cells after removing dead cells. Data from 4 patients (0 months [0M], 6M, and 12M) include a total of 37,314 cells: case 1 (17,510 cells), case 2 (8,067 cells), case 3 (8,201 cells), and case 5 (3,536 cells). Cells were clustered into 19 groups, color-coded based on metadata. (**B**) Dot plot showing expression levels of key genes in each cluster. Dot color represents scaled expression, and size indicates the percentage of cells expressing each gene. (**C**) Visualization of cell distributions at 0M (10,187 cells), 6M (14,095 cells), and 12M (13,032 cells). (**D**) Changes in cell cluster proportions over time (0M, 6M, 12M).

Unsupervised clustering of transcriptomic data identified 19 distinct cell populations (Figure 3A). Epithelial cells were defined by high *EPCAM* expression, while immune cell populations, including CD4^+^ and CD8^+^ T cells, were identified based on *CD3E* and *PTPRC* expression patterns (Figure 3B). Relative cell type abundances were quantified across the 3 time points (Figure 3C). Overall, the proportions of epithelial cells, B cells, and plasma cells were similar between 0M and 6M (Supplemental Figure 2, A and B). However, in cases 1 and 3, the proportion of epithelial cells increased markedly at 12M compared with baseline (Figure 3D and Supplemental Figure 3, A and B). Notably, the frequencies of other immune populations, including total T and B cells, remained relatively stable throughout the treatment period. These findings indicate that prolonged GLP-2 treatment is associated with an expansion of the epithelial cell compartment in the small intestine, consistent with enhanced mucosal remodeling and regeneration.

### GLP-2 therapy modulates the intestinal immune cell landscape with selective changes in T cell subsets.

**Figure 4:**
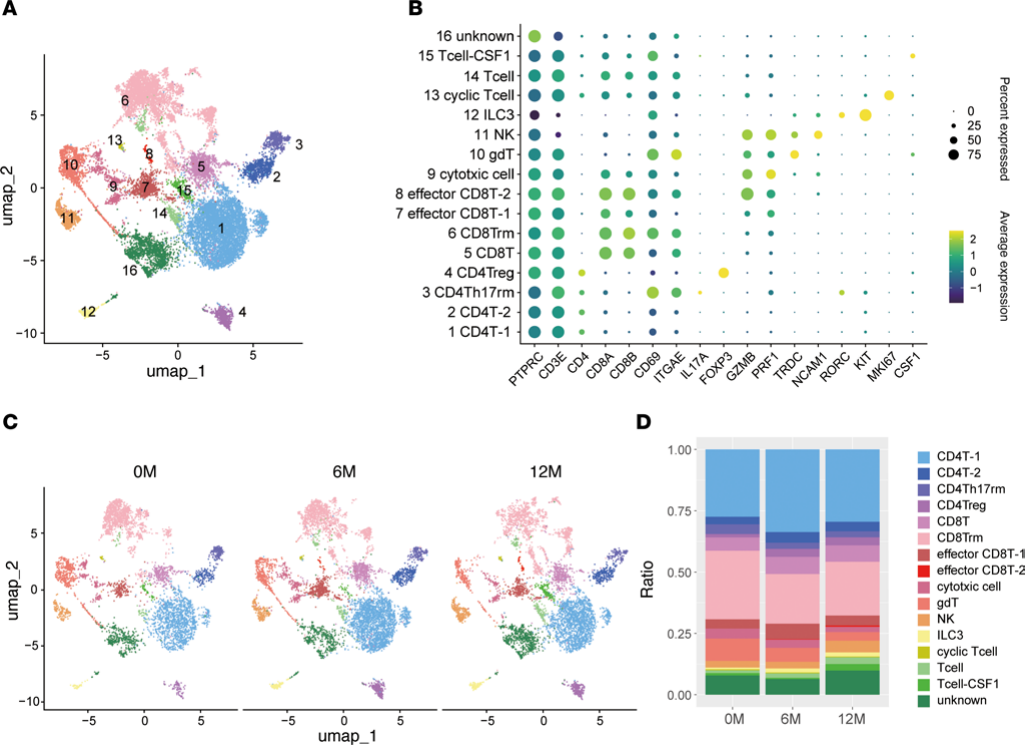
GLP-2 therapy modulates the intestinal immune cell landscape with selective changes in T cell subsets. (**A**) Uniform manifold approximation and projection (UMAP) visualization of immune cells identified using *PTPRC*, with B cells, plasma cells, and myeloid cells excluded. Data from 4 patients at 0 months (0M), 6M, and 12M include a total of 17,736 cells: case 1 (7,769 cells), case 2 (3,915 cells), case 3 (4,161 cells), and case 5 (1,891 cells). Cells were clustered into 16 groups, color-coded by metadata. (**B**) Dot plot showing expression levels of key genes in immune cell clusters. (**C**) Cell distributions at 0M (4,937 cells), 6M (6,653 cells), and 12M (6,146 cells). (**D**) Changes in immune cell clusters at 0M, 6M, and 12M. ILC, innate lymphoid cell; NK, natural killer.

To delineate changes in the immune cell landscape during GLP-2 analog therapy, we identified immune cells by *PTPRC* expression and further resolved them into 16 distinct clusters using unsupervised clustering (Figure 4A). CD4^+^ T cell populations were distributed across the CD4T-1, CD4T-2, regulatory T cell (Treg), and Th17 resident memory clusters, while CD8^+^ T cells were classified into CD8T, CD8T resident memory, effector CD8T-1, effector CD8T-2, cycling T, and general T cell clusters (Figure 4B). Innate immune subsets were also identified, including cytotoxic cells, T cell receptor γδ^+^ (TCRγδ^+^) cells, NK cells, and group 3 ILCs, characterized by the expression of *TRDC*, *NCAM1*, and *RORC*, respectively. Of note, cycling T cells expressed elevated levels of *MKI67*, indicative of proliferative activity (Supplemental Figure 4A). We next assessed the distribution of immune clusters across patients and time points (Supplemental Figure 4, B–E). The relative proportions of TCRγδ^+^ cells, ILC3s, and NK cells remained largely stable between 0M and 6M (Figure 4, C and D, and Supplemental Figure 4, D and E). However, a trend toward increased ILC3s’ abundance was observed at 6M compared with baseline. Given the limited changes among innate subsets, our subsequent analyses focused on the temporal dynamics of CD4^+^ and CD8^+^ T cell populations, which may be more responsive to GLP-2–mediated mucosal modulation.

### GLP-2 analog treatment selectively expands Tregs and activates growth-associated signaling pathways in the CD4^+^ compartment.

**Figure 5:**
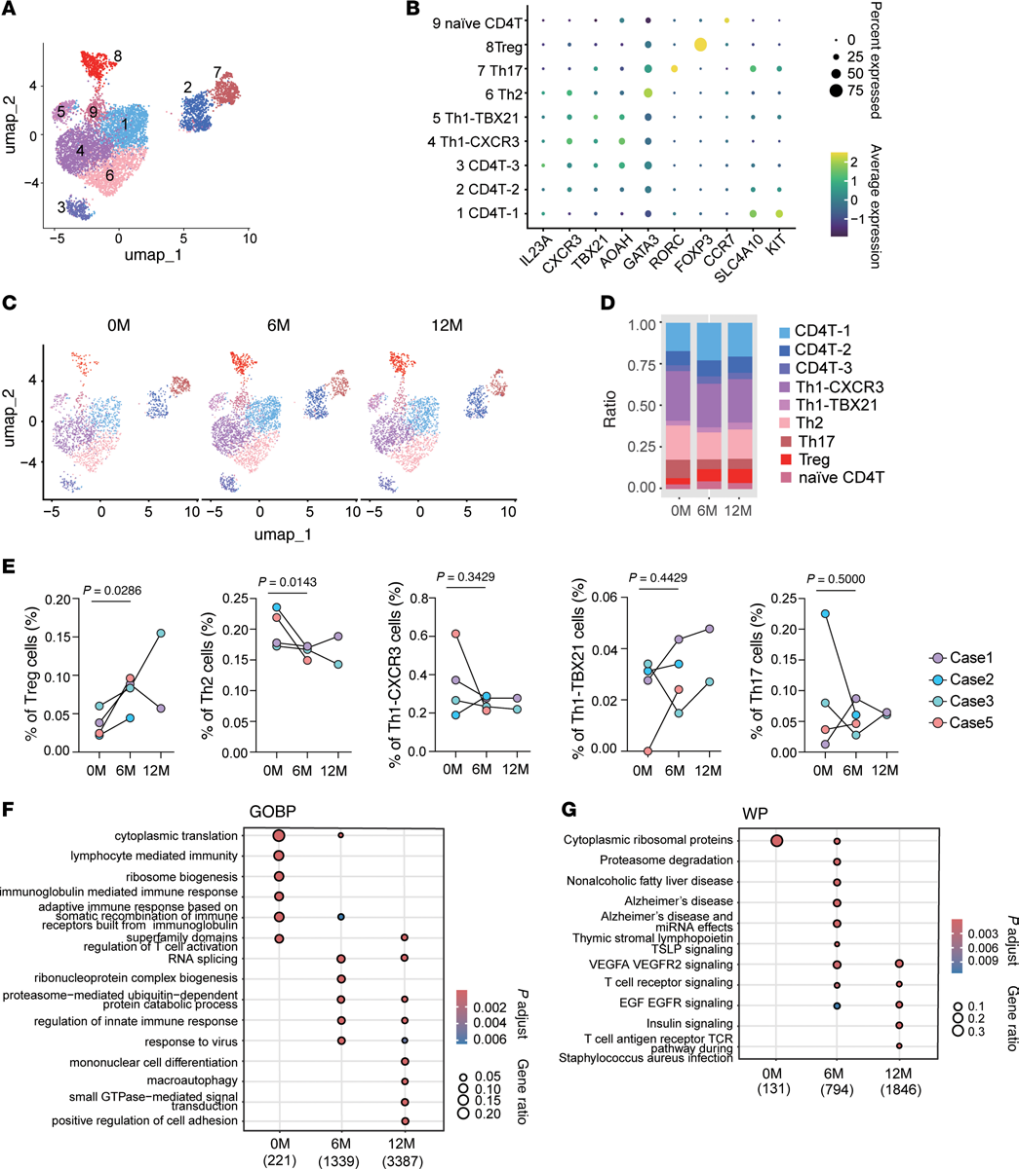
GLP-2 analog treatment selectively expands Tregs and activates growth-associated signaling pathways in the CD4^+^ compartment. (**A**) Uniform manifold approximation and projection (UMAP) visualization of CD4^+^ T cells identified using *CD4*. Data from 4 patients at 0 months (0M), 6M, and 12M include a total of 7,093 cells: case 1 (2,990 cells), case 2 (1,697 cells), case 3 (1,703 cells), and case 5 (703 cells). Cells were clustered into 9 groups, color-coded by metadata. (**B**) Expression patterns of key genes in CD4^+^ T cell clusters. Each dot represents a gene, where color intensity indicates the scaled expression level in each cluster, and dot size represents the percentage of cells expressing the gene. (**C**) Cell distributions at 0M (1,772 cells), 6M (2,916 cells), and 12M (2,405 cells). (**D**) Proportion of each cluster in CD4^+^ T cells at 0M, 6M, and 12M. (**E**) The percentage of Th1-CXCR3 cells, Th1-TBX21 cells, T regulatory cells (Tregs), Th17 cells, and Th2 cells at 0M, 6M, and 12M. (**F**) Comparison of differentially expressed gene (DEG) sets related to Gene Ontology (GO) Biological Process (BP) across 0M, 6M, and 12M using compareCluster. Significantly enriched GO terms (*y*-axis) with *P* < 0.01 and *q* < 0.05 are visualized as dot plots. (**G**) Comparison of DEG sets related to biological pathways based on WikiPathways (WP) across 0M, 6M, and 12M using compareCluster. Significantly enriched pathways (*y*-axis) with *P* < 0.05 and *q* < 0.2 are visualized as dot plots. (**F** and **G**) Numbers below the column name represent the number of genes related to GOBP (**F**) or WP (**G**) for each cluster. Dot size represents the gene ratio (proportion of DEGs), and color indicates the adjusted *P* value. The 2-tailed paired Student’s *t* test was used to compare cluster proportions (**E**). compareCluster analysis was performed using Over-Representation Analysis (ORA) based on a hypergeometric test. Multiple-testing correction was applied using the Benjamini-Hochberg (BH) method to control the false discovery rate (FDR) (**F** and **G**).

Single-cell transcriptomic profiling of CD4^+^ T cells identified 9 distinct clusters based on gene expression signatures (Figure 5A). Naive CD4^+^ T cells were marked by *CCR7* expression, while Tregs were defined by high *FOXP3* expression. Th2 and Th17 cells were characterized by *GATA3* and *RORC* expression, respectively. Th1 subsets were split into 2 clusters: Th1-CXCR3 and Th1-TBX21, based on predominant expression of *CXCR3* and *TBX21*. Additional CD4^+^ clusters (CD4-1, CD4-2, CD4-3) included a subset with high *SLC4A10* and *KIT* expression (Figure 5B and Supplemental Figure 5A).

Quantitative analysis revealed a consistent increase in the proportion of Tregs within the CD4^+^ population across patients during treatment, while Th2 cells declined over the same period (Figure 5, C–E, and Supplemental Figure 5, B and C). In contrast, the frequencies of Th1-CXCR3, Th1-TBX21, and Th17 cells remained relatively unchanged. Gene Ontology (GO) enrichment analysis revealed upregulation of pathways involved in vascular endothelial growth factor (VEGF) signaling, TCR signaling, and epidermal growth factor (EGF) signaling at both 6M and 12M (Figure 5, F and G). We then performed separate GO enrichment analyses for Treg and Th2 cell populations (Supplemental Figure 5, D and E). Prior to treatment, Th2 cells were enriched for pathways related to adaptive immune response and lymphocyte-mediated immunity. In contrast, Tregs posttreatment showed enrichment for pathways such as positive regulation of tolerance induction and regulation of CD4^+^ αβ T cell proliferation. These data suggest that GLP-2 therapy enhances immune tolerance pathways in Tregs while dampening immune activation in Th2 cells.

### Diverse CD8^+^ T cell subset dynamics reflect patient-specific responses to GLP-2 analog therapy.

**Figure 6:**
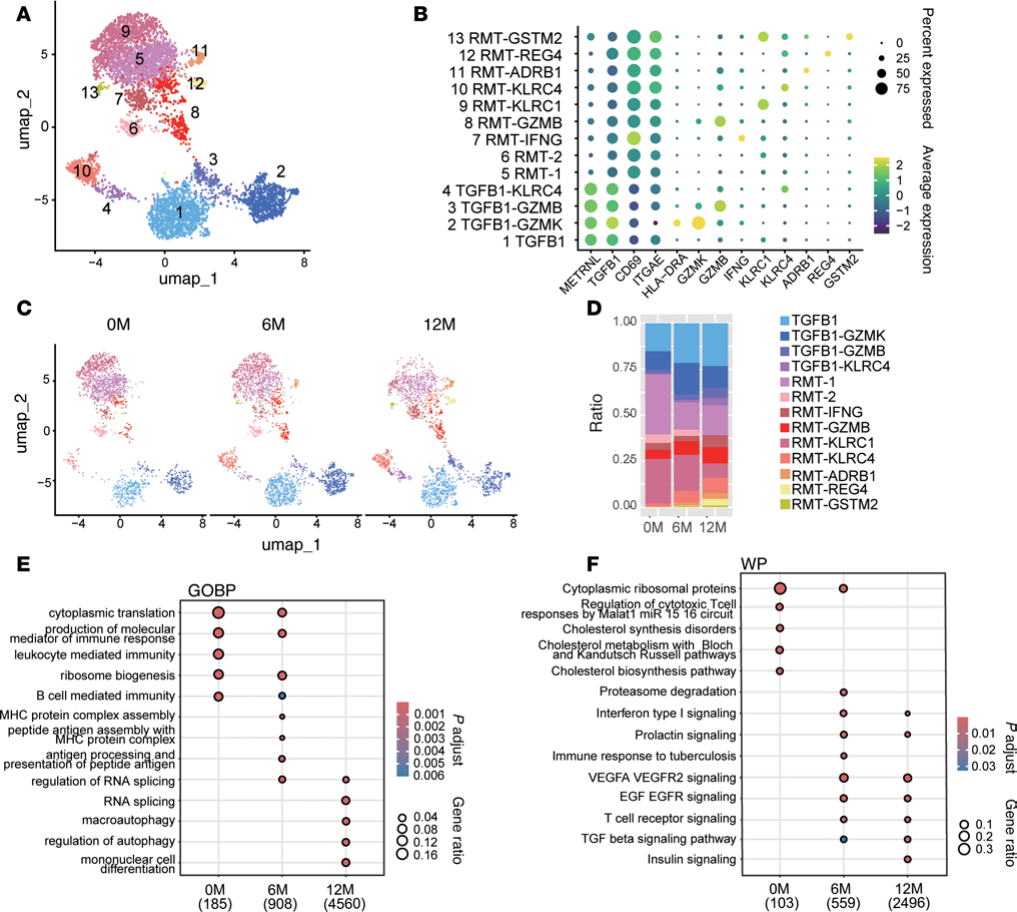
Diverse CD8^+^ T cell subset dynamics reflect patient-specific responses to GLP-2 analog therapy. (**A**) Uniform manifold approximation and projection (UMAP) visualization of CD8^+^ T cells identified using *CD8A* and *CD8B* markers. Data from 4 patients at 0 months (0M), 6M, and 12M include a total of 5,904 cells: case 1 (2,486 cells), case 2 (1,300 cells), case 3 (1,472 cells), and case 5 (646 cells). Cells were clustered into 13 groups, color-coded by metadata. (**B**) Expression patterns of key genes in CD8^+^ T cell clusters. Each dot represents a gene, where color intensity indicates the scaled expression level in each cluster, and dot size represents the percentage of cells expressing the gene. (**C**) Cell distributions at 0M (1,744 cells), 6M (2,185 cells), and 12M (1,975 cells). (**D**) Proportion of each cluster in CD8^+^ T cells at 0M, 6M, and 12M. (**E**) Comparison of differentially expressed gene (DEG) sets related to Gene Ontology (GO) Biological Process (BP) across 0M, 6M, and 12M using compareCluster. Significantly enriched GO terms (*y*-axis) with *P* < 0.01 and *q* < 0.05 are visualized as dot plots. (**F**) Comparison of DEG sets related to biological pathways based on WikiPathways (WP) across 0M, 6M, and 12M using compareCluster. Significantly enriched pathways (*y*-axis) with *P* < 0.05 and *q* < 0.2 are visualized as dot plots. (**E** and **F**) Numbers below the column name represent the number of genes related to GOBP (**E**) or WP (**F**) for each cluster. Dot size represents the gene ratio (proportion of DEGs), and color indicates the adjusted *P* value. compareCluster analysis was performed using Over-Representation Analysis (ORA) based on a hypergeometric test. Multiple testing correction was applied using the Benjamini-Hochberg (BH) method to control the false discovery rate (FDR) (**E** and **F**).

Within the CD8^+^ T cell compartment, 13 transcriptionally distinct clusters were identified (Figure 6A). These included resident memory T cells (RMTs), defined by high *CD69* and *ITGAE* expression, spanning RMT-1, RMT-2, RMT-IFNG, RMT-GZMB, RMT-KLRC1, RMT-KLRC4, RMT-ADRB1, RMT-REG4, and RMT-GSTM2 subsets. Additionally, clusters characterized by low *CD69* and high *TGFB1* expression were grouped as TGFB1-like populations, including TGFB1, TGFB1-GZMK, TGFB1-GZMB, and TGFB1-KLRC4 (Figure 6B and Supplemental Figure 6A).

While no single CD8^+^ subset exhibited consistent enrichment across all cases, patient-specific changes were observed. For instance, RMT-KLRC4 increased in case 1, TGFB1 in case 2, RMT-IFNG in case 3, and TGFB1-GZMK in case 5 (Supplemental Figure 6, B and C). Notably, RMT-KLRC4 showed a general increase at both 6M and 12M across patients (Figure 6, C and D). GO analysis and WikiPathway analysis of CD8^+^ T cells also revealed enrichment in VEGF, EGF, TCR, TGF-β, and insulin signaling pathways following treatment (Figure 6, E and F). Collectively, these findings highlight a treatment-associated increase in Treg abundance within the CD4^+^ compartment, while CD8^+^ T cell dynamics were more heterogeneous. Nonetheless, both populations exhibited convergent upregulation of signaling pathways associated with tissue remodeling, immune modulation, and homeostasis.

### GLP-2 therapy promotes nutrient-absorptive epithelial lineages while suppressing immune-associated enterocyte subtypes.

**Figure 7:**
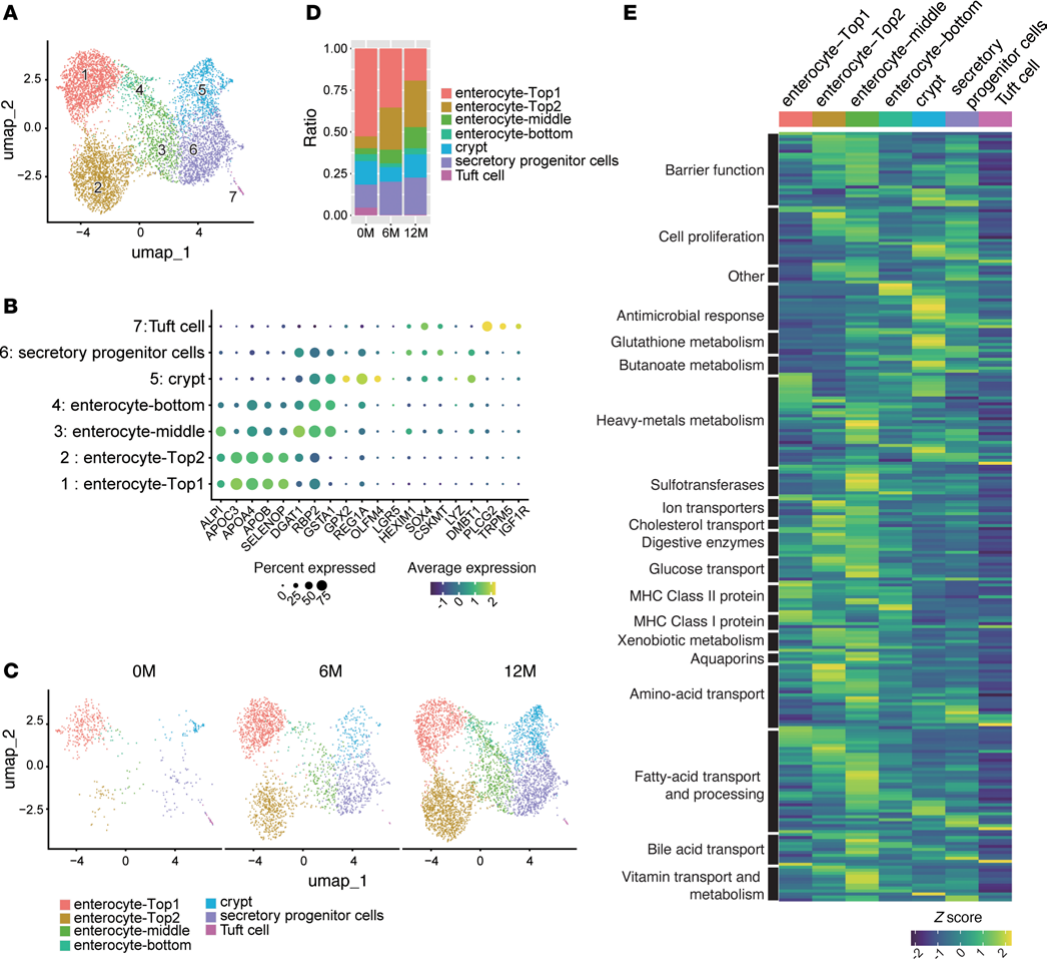
GLP-2 therapy promotes nutrient-absorptive epithelial lineages while suppressing immune-associated enterocyte subtypes. (**A**) Uniform manifold approximation and projection (UMAP) embedding of epithelial cells identified by *EPCAM* expression from the total cell population. Cells from all 4 patients across 3 time points (0 months [0M], 6M, and 12M) are included, with a total of 6,636 cells (5,112 from case 1, 244 from case 2, 936 from case 3, and 344 from case 5). Cells are clustered into 7 groups, colored by metadata as indicated in the figure. (**B**) Expression patterns of key marker genes for each cluster. Each dot represents a gene, where color saturation indicates scaled expression levels, and dot size represents the percentage of cells expressing that gene. (**C**) UMAP embedding of epithelial cells across 3 time points: 0M, 6M, and 12M (505 cells at 0M, 2,076 cells at 6M, and 4,055 cells at 12M). (**D**) Proportions of epithelial cell clusters at 0M, 6M, and 12M. (**E**) Heatmap of *Z*-score normalized gene expression for key intestinal mucosal pathways across clusters. Columns represent clusters (indicated by color and label), and rows represent genes categorized by pathway. To compare gene expression between clusters, the Wilcoxon rank-sum test was used.

We analyzed epithelial cells to assess changes following GLP-2 treatment and identified 7 distinct enterocyte clusters based on gene expression and zonation-specific markers (22, 23) (Figure 7, A–C, and Supplemental Figure 7, A–C). Enterocyte-Top1 and -Top2 clusters, localized to the villus tip, were defined by high expression of *ALPI*, *APOC3*, *APOA4*, *APOB*, and *SELENOP*. Enterocyte-middle and enterocyte-bottom clusters, positioned in the mid- and basal villus, were marked by *DGAT1*, *RBP2*, and *GSTA1*. A crypt-associated cluster, enriched in *GPX2* and *REG1A*, was designated as the crypt cluster. Secretory progenitor cells were defined by *HEXIM1*, *SOX4*, *CSKMT*, and *DMBT1*, while the tuft cell cluster expressed *PLCG2*, *TRPM5*, and *IGF1R*.

As expected, total epithelial cell numbers increased following GLP-2 treatment. Analysis of relative cluster proportions revealed a decrease in enterocyte-Top1 and an increase in enterocyte-Top2 posttreatment (Figure 7, C and D, and Supplemental Figure 8, A and B). The enterocyte-middle and -bottom clusters remained stable, whereas crypt and tuft cell clusters declined. Patient-specific analysis showed increased enterocyte-Top2 and middle clusters in cases 2, 3, and 5 but not in case 1.

GO analysis indicated that enterocyte-Top1 was enriched in immune-related pathways, including T cell activation, leukocyte adhesion, and TCR signaling (Supplemental Figure 8C). In contrast, enterocyte-Top2 was associated with pathways related to nutrient processing, such as lipid transport, glycerolipid metabolism, and intestinal absorption, suggesting distinct functional specializations (Supplemental Figure 8D). To assess these roles more directly, we evaluated functional gene expression across clusters (Figure 7E and Supplemental Figure 9). Barrier integrity and nutrient-processing genes — covering ion transport, amino acid and glucose transport, cholesterol and fatty acid metabolism, digestive enzymes, and bile acid and vitamin transport — were enriched in enterocyte-Top2 and middle clusters. Conversely, MHC class I/II genes were predominantly expressed in enterocyte-Top1, supporting its role in immune modulation. The crypt cluster showed enrichment in glutathione metabolism, butanoate metabolism, cell proliferation, and antimicrobial responses. Notably, VEGFA and EGFR were enriched in enterocyte-Top2 and middle clusters, whereas IGF1R was specific to tuft cells. These findings suggest that the enterocyte-Top1 cluster is primarily involved in immune responses and MHC class I and II expression, whereas enterocyte-Top2 and middle clusters are more specialized for nutrient absorption, including amino acid, glucose, and fatty acid transport, as well as cholesterol transport. The crypt cluster was enriched in antimicrobial responses and metabolic pathways, such as glutathione and butanoate metabolism.

### Longitudinal gene expression profiling uncovers coordinated enhancement of absorptive function and barrier integrity in response to GLP-2.

**Figure 8:**
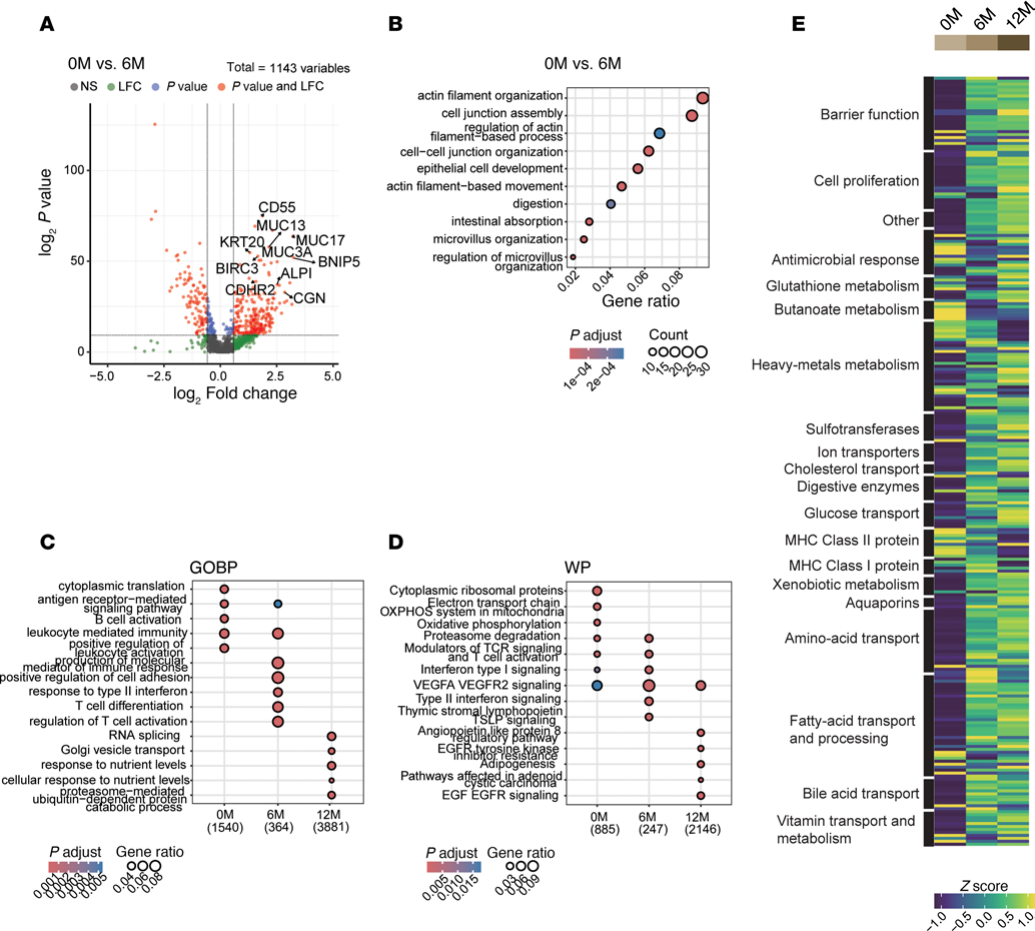
Longitudinal gene expression profiling uncovers coordinated enhancement of absorptive function and barrier integrity in response to GLP-2. (**A**) Volcano plot showing differentially expressed genes (DEGs) in epithelial cells at 0 months (0M) versus 6M. The *x*-axis represents Log2 fold change (LFC), and the *y*-axis represents –Log10 (*P* value). Vertical dashed lines indicate a threshold of |LFC| > 0.58, and the horizontal dashed line indicates a *P* value cutoff of 5 × 10^–10^. (**B**) Gene Ontology (GO) pathway enrichment analysis of DEGs between 0M and 6M. Genes with LFC > 0.58 that were upregulated at 6M were analyzed. Dot plots display significantly enriched biological processes (BPs) (*P* < 0.01, *q* < 0.05), with dot size indicating gene count and color representing the adjusted *P* value. (**C**) GOBP enrichment analysis of DEGs across 0M, 6M, and 12M using compareCluster. (**D**) Differential gene expression analysis of biological pathways based on WikiPathways (WP) across 0M, 6M, and 12M using compareCluster. (**C** and **D**) Numbers below the column name represent the number of genes related to GOBP (**C**) or WP (**D**) for each cluster. Dot plots show significantly enriched GO terms (*P* < 0.01, *q* < 0.05) (**C**) or pathways (*P* < 0.05, *q* < 0.2) (**D**), with dot size representing the gene ratio (proportion of DEGs) and color indicating the adjusted *P* value. (**E**) Heatmap of *Z*-score normalized gene expression for key intestinal mucosal pathways across time points. Columns represent clusters (indicated by color and label), and rows represent genes categorized by pathway. GO analysis and compareCluster analysis were performed using Over-Representation Analysis (ORA) based on a hypergeometric test (**B**–**D**). Multiple-testing correction was performed using the Benjamini-Hochberg (BH) method to control the false discovery rate (FDR) (**C** and **D**). To compare gene expression between clusters, the Wilcoxon rank-sum test was used (**E**).

We next examined transcriptomic changes over the treatment period. Differential gene expression analysis showed significant upregulation of genes involved in differentiation (*KRT20*, *BNIP5*), antiapoptotic signaling (*BIRC3*), barrier function (*MUC13*, *MUC17*), immune regulation (*CD55*), nutrient processing (*GLS*, *TMC5*), cell movement (*MYH14*), and tight junction (*ACTN4*) at 6M and 12M compared with 0M (Figure 8, A and B, and Supplemental Figure 10, A and B). GO analysis revealed that antigen receptor-mediated signaling was enriched at baseline (0M), while nutrient response pathways predominated at 12M (Figure 8C). WikiPathway analysis supported these trends, with T cell activation enriched at 0M and 6M and both VEGFA/VEGFR2 and EGF/EGFR signaling enriched at 6M and 12M (Figure 8D).

To validate these functional shifts, we reassessed expression of key gene sets over time (Figure 8E and Supplemental Figure 11). Barrier and nutrient absorption genes increased at 6M and 12M, while immune-related genes, including MHC class I and II, were downregulated. Collectively, these findings indicate that GLP-2 analog treatment promotes the expansion of enterocyte-Top2 and middle clusters specialized in nutrient uptake, while reducing enterocyte-Top1 cells associated with immune activity.

**Figure 9:**
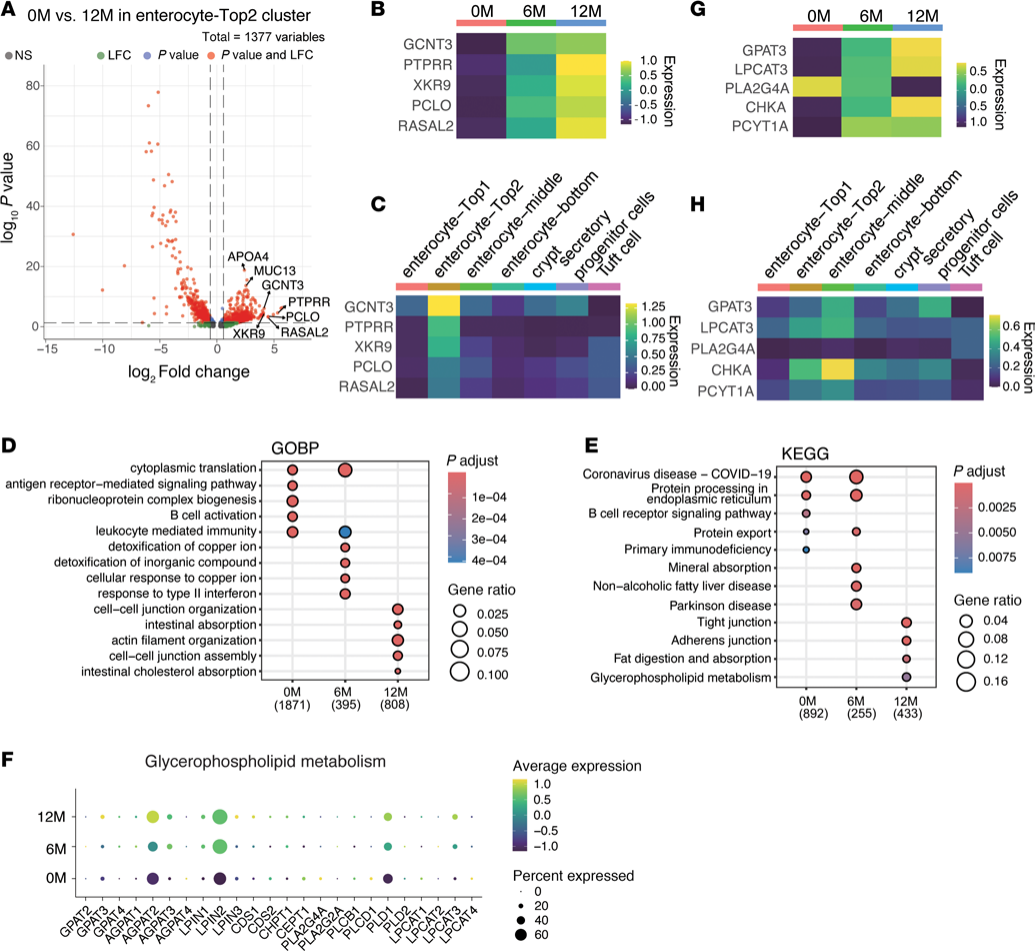
Longitudinal gene expression analysis of enterocyte-Top2 cluster reveals functional reprogramming in response to GLP-2. (**A**) Volcano plot showing differentially expressed genes (DEGs) in enterocyte-Top2 cluster at 0 months (0M) versus 12M. The *x*-axis represents Log2 fold change (LFC), and the *y*-axis represents –Log10 (*P* value). Vertical dashed lines indicate a threshold of |LFC| > 0.58, and the horizontal dashed line indicates a *P* value cutoff of 0.05. (**B** and **C**) Heatmap of *Z*-score normalized gene expression for DEGs shown in **A**, across time points (**B**) and clusters (**C**). (**D** and **E**) CompareCluster analysis of DEGs in enterocyte-Top2 cluster across 0M, 6M, and 12M. Rows represent Gene Ontology (GO) Biological Process (BP) (**D**) and Kyoto Encyclopedia of Genes and Genomes (KEGG) pathways (**E**). Dot plots show significantly enriched GO terms or pathways (*P* < 0.01, *q* < 0.05), with dot size indicating the gene ratio (proportion of DEGs) and color indicating the adjusted *P* value. Numbers below the column name indicate the number of genes related to the pathways for each cluster. (**F**) Gene expression related to glycerophospholipid metabolism in enterocyte-Top2 cluster across time points. Each dot represents a gene, color intensity indicates the scaled expression level in each cluster, and dot size represents the percentage of cells expressing the gene. (**G** and **H**) Heatmap of *Z*-score normalized expression of genes related to glycerophospholipid metabolism across time points (**G**) and clusters (**H**). compareCluster analysis was performed using Over-Representation Analysis (ORA) based on a hypergeometric test (**D** and **E**). Multiple-testing correction was performed using the Benjamini-Hochberg (BH) method to control the false discovery rate (FDR) (**D** and **E**). To compare gene expression between clusters, the Wilcoxon rank-sum test was used (**B**, **C**, **G**, and **H**).

In addition, we analyzed gene expression changes within the enterocyte-Top2 population over the treatment course (Figure 9A). *MUC13*, *GCNT3* (involved in mucin regulation and barrier function), *APOA4* (lipid absorption), *PTPRR* (link to immunosuppressive signaling), *XKR9* (apoptosis-related gene), and *PCLO* and *RASAL2* (negative regulators of RAS signaling) gene expressions were upregulated at 12M after GLP-2 agonist treatment (Figure 9, B and C). These genes were specifically and highly expressed within the enterocyte-Top2 population. GO enrichment analysis of this population revealed increased activity in pathways related to intestinal absorption, cell-cell junction assembly, and intestine cholesterol absorption at 12M after treatment (Figure 9D). Consistently, KEGG pathway analysis demonstrated enrichment in pathways associated with tight junction, fat digestion and absorption, and glycerophospholipid metabolism (Figure 9E). These data support the conclusion that GLP-2 analog treatment not only promotes expansion of enterocyte-Top2 population but also enhances their functional gene programs related to nutrient uptake and epithelial barrier integrity. Furthermore, we examined SCFA-induced gene expression in epithelial cells, particularly within the enterocyte-Top2 cluster. SCFA is known to induce glycerophospholipid metabolism, which maintains membrane integrity and barrier function, so we further examined this pathway. Expression levels of key genes in glycerophospholipid pathway, such as *GPAT3*, *LPCAT3*, *PLA2G4A*, and *CHKA*, were progressively upregulated throughout treatment (Figure 9F). Notably, these genes, particularly *LPCAT3*, *CHKA*, and *PCYT1A*, were enriched in enterocyte-Top2 and middle clusters (Figure 9, G and H). Longitudinal analysis verified sustained upregulation of these genes over the treatment period (Figure 9H). These findings support a model in which increased SCFA level enhances glycerophospholipid metabolism, reinforcing membrane stability and tight junction integrity in enterocyte-Top2 cluster, thus linking microbiome metabolism to epithelial function.

**Figure 10:**
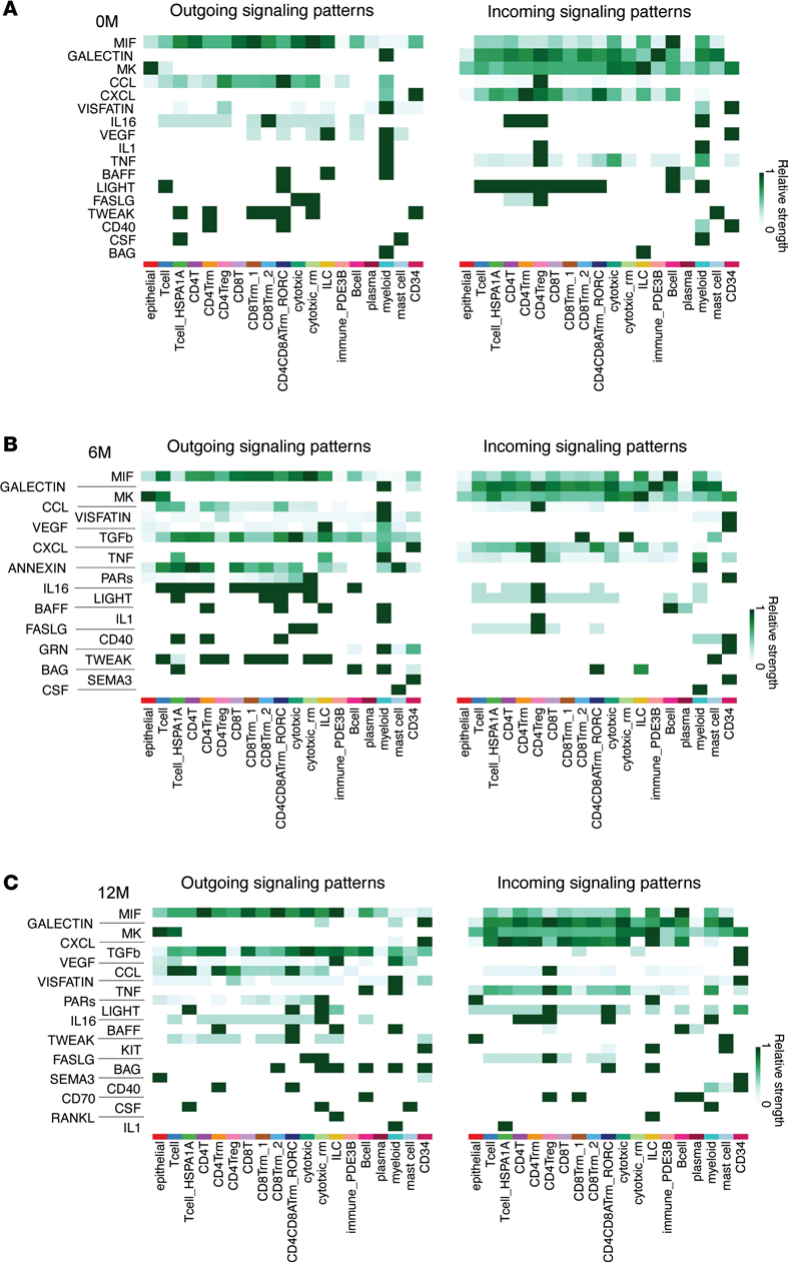
Changes in ligand–receptor interactions between clusters reveal enhanced intercellular communication following GLP-2 analog treatment. (**A**–**C**) Heatmaps showing outgoing signaling patterns (left panels) and incoming signaling patterns (right panels) for each cluster at 0 months (0M) (**A**), 6M (**B**), and 12M (**C**). Columns represent cell clusters (indicated by color and label), and rows represent signaling pathways. Relative interaction strength is shown on the scale bars.

To further explore potential mediators of enterocyte-Top2 cell expansion, we conducted ligand–receptor interaction analysis using our single-cell transcriptomics dataset (Figure 10, A–C). At 0M, key outgoing signaling pathways included MIF, GALECTIN, MK, CCL, and CXCL, while incoming signaling was generally weak but exhibited connectivity among immune cell subsets. By 6M, additional outgoing pathways, such as TGF-β, ANNEXIN, PARs, GRN, and SEMA3, emerged alongside those observed at baseline, indicating progressive complexity in the outgoing signaling landscape. Concurrently, incoming signaling strength increased, particularly within regulatory and memory T cell populations. At 12M, the intercellular signaling network further expanded, with dominant outgoing pathways including TGF-β, VEGF, PARs, KIT, RANKL, and CD70. The overall increase in incoming signaling strength at this time point supports a robust enhancement of intercellular communication following GLP-2 analog treatment. Several signaling pathways, MIF, GALECTIN, MK, CXCL, VEGF, CCL, VISFATIN, IL16, TNF, BAFF, LIGHT, FASLG, TWEAK, CD40, CSF, BAG, and IL1, were consistently detected across all time points, suggesting they participate in maintaining basal immune–epithelial interactions. In contrast, TGF-β, PARs, KIT, SEMA3, CD70, and RANKL were uniquely upregulated at 12M, indicating the emergence of specific biological processes, including tissue remodeling (TGF-β), neuro-immune signaling (SEMA3), and enhanced adaptive immunity (CD70, RANKL).

**Figure 11:**
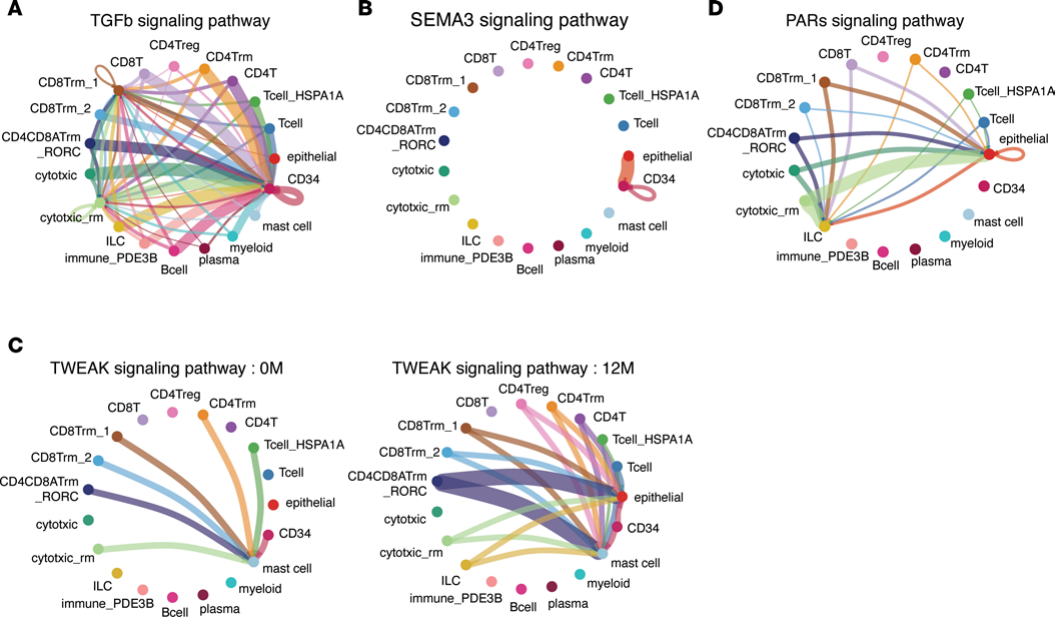
GLP-2 analog treatment enhances communication between epithelial, immune, and CD34^+^ cells. (**A**–**D**) Circle plots showing intercellular signaling networks. The TGF-β (**A**), SEMA3 (**B**), and PARs (**D**) signaling pathways were newly detected at 12 months (12M). The TWEAK signaling pathway (**C**) was upregulated at 12M compared with 0M. Arrow colors indicate the source (sender) clusters of the signaling, and arrow widths represent the relative signaling strength.

To further dissect cellular crosstalk, we examined interactions between immune and nonimmune cell populations, including epithelial cells and CD34^+^ cells. Immune cells broadly upregulated TGF-β signaling as senders, while CD34^+^ cells showed corresponding receptor expression, suggesting they act as primary receivers (detected at 12M) (Figure 11A). Additionally, SEMA3 (detected at 12M), produced by epithelial cells, showed strong correlation with CD34^+^ cells, as did upregulated VEGF and VISFATIN signaling pathways (Figure 11B and Supplemental Figure 12, A and B). These interactions are consistent with a GLP-2–induced program supporting vascular regeneration. At 12M, epithelial cells also upregulated outgoing signals, such as TWEAK and PARs (detected at 12M), toward immune populations, supporting roles in barrier maintenance and tissue repair (Figure 11, C and D). Overall, our data suggest that GLP-2 analog treatment enhances communication between epithelial, immune, and CD34^+^ cells, which foster an immune-suppressive, tissue-protective environment that reinforces intestinal barrier integrity.

## Discussion

GLP-2 analogs are clinically effective in promoting intestinal adaptation in patients with SBS, reducing dependence on parenteral nutrition. While their trophic effects on the intestinal mucosa are well established (17, 18), the mechanisms by which GLP-2 therapy modulates epithelial cell differentiation and intestinal immune compartments in humans have remained largely undefined. In this study, we combined longitudinal single-cell transcriptomics with microbiome profiling to elucidate the cellular and molecular remodeling events induced by GLP-2 analog treatment in SBS. Our findings offer mechanistic insights into how GLP-2 promotes nutrient absorption and orchestrates immune adaptation in the human intestine.

Clinically, patients showed improvements in nutritional status, including weight gain, reduced parenteral support, and increased levels of citrulline and lipase, consistent with enhanced absorptive function. These improvements were accompanied by histological increases in villus height and crypt depth, reinforcing the known trophic effects of GLP-2 on mucosa. These physiological adaptations aligned with decreased stool output and reduced need for parenteral nutrition, underscoring the therapeutic impact of GLP-2 analog in SBS management.

Despite clinical improvements, microbiome responses to GLP-2 analog were heterogeneous across the patients, echoing prior findings on interindividual variability in upper gastrointestinal microbiome composition (24–27). Microbial analysis revealed increased α-diversity over time, although taxonomic changes were patient specific. Functional inference, however, indicated upregulation of pathways related to SCFA biosynthesis, including acetate and butanoate. These metabolites are known to enhance epithelial barrier integrity and regulate immune responses, suggesting that GLP-2 drives a functional, rather than compositional, restructuring of the microbiome. SCFA signaling has also been linked to induction of glycerophospholipid metabolism, a pathway we found upregulated in absorptive enterocyte clusters. These findings support a model in which GLP-2–induced microbial changes promote epithelial membrane stability and tight junction integrity via SCFA-mediated signaling.

Immunologically, we observed an increase in Tregs and a concurrent decrease in Th2 cells across patients. This immunological shift likely reflects improved epithelial barrier function and reduced antigen exposure (28–30), as well as increased microbial production of immunomodulatory metabolites, such as SCFA (31–39). While GLP-2 receptors are not expressed in immune cells (40), these changes are plausibly mediated by downstream effects of epithelial and stromal remodeling (41, 42). Notably, ILC3s, which play a critical role in maintaining epithelial homeostasis through cytokines such as IL-22 (43–54), showed a subtle but consistent increase during the treatment. Together, these findings suggest that GLP-2 analog had indirect but significant effects on mucosal immunity, positioning the hormone as modulator of both structural and immunological adaptation in the gut.

Concurrently, scRNA-Seq of intestinal biopsies revealed that GLP-2 analog treatment promotes a shift toward absorptive phenotypes. Specifically, we observed expansion of enterocyte-Top2 and middle clusters enriched for genes involved in lipids, amino acids, and vitamin transport along with upregulation of barrier function markers (22, 23). In contrast, enterocyte-Top1 cells characterized by high MHC class I/II expression declined suggesting a shift away from epithelium–immune interactions toward a more absorptive epithelial phenotype (55, 56). Importantly, GLP-2 receptors were not expressed in these absorptive enterocytes, implying that trophic effects are mediated through paracrine signaling from GLP-2–expressing subepithelial myofibroblasts or neural cells via IGF-1 and EGF pathways, consistent with prior murine studies (2, 3, 5, 11, 57–59). Indeed, *IGF1R* was notably enriched in tuft cells (60), while *EGFR* expression was prominent in enterocyte-Top2 clusters, consistent with previous reports (61–64). Our findings suggest that GLP-2 analogs may enhance the development of enterocyte-Top2 clusters through EGFR signaling.

This study has limitations. The sample size was small, was restricted to male patients, and lacked certain stromal and innate immune populations, such as fibroblasts, mesenchymal cells, and granulocytes. Additionally, ligand–receptor and pathway activity were inferred computationally and will require experimental validation. Nonetheless, the longitudinal single-cell approach provides a view of epithelial, immune, and microbial remodeling in human SBS during GLP-2 therapy. Despite these constraints, our longitudinal design and integrative single-cell approach offer a comprehensive view of the cellular ecosystem remodeling that occurs during GLP-2 therapy in humans.

In conclusion, GLP-2 analog treatment in SBS orchestrated a coordinated remodeling of the intestinal ecosystem, promoting absorptive epithelial differentiation, regulatory immune adaptation, and SCFA-associated microbial function. These findings provide mechanistic insights into GLP-2–driven intestinal rehabilitation and may inform therapeutic strategies for malabsorptive and inflammatory intestinal disorders.

## Methods

See the Supplemental Methods for more information.

### Sex as a biological variable.

Our study involved 5 male participants due to the limited availability of eligible patients.

However, the underlying mechanisms targeted in GLP-2 analog are conserved in female and used clinically in both male and female individuals, and thus the findings are expected to be relevant to both sexes.

### Statistics.

All graphs show mean ± SD unless otherwise stated. *P* values less than 0.05 were considered significant.

### Study approval.

All human samples were obtained from individuals providing written informed consent following protocols approved by the Keio University IRB.

### Data availability.

All data are available in the main text or the supplemental material. Individual data points for each figure are available in the Supporting Data Values file.

16S-rRNA-Seq data are available on the NCBI BioProject with the entry number PRJDB20353.

ScRNA-Seq data are available on the NCBI GEO with the entry number GSE294205.

## Author contributions

YK, Y Yamada, and TS conceptualized and designed the study. Investigation was performed by YK, AC, AT, MH, AS, IK, YA, and SS (human tissue preparation and analysis) and MS, AK, T Kumagai, HD, YS, YI, KS, SY, YM, HK, M Kano, Mototoshi Kato, Y Yoshimatsu, KT, KO, T Kanai, NH, Motohiko Kato, T Kumagai and T Kuroda (clinical data analysis). YK and HT performed pathological data analysis. ScRNA-Seq and microbiome analyses, as well as data visualization, were conducted by YK and KM. Funding acquisition was carried out by Y Yamada and TS. AF, Y Yamada, and TS supervised the study. YK, KM, and TS wrote the original draft. Review and editing were performed by JM, CCH, AF, Y Yamada, and TS. All authors reviewed and approved the manuscript.

## Supplementary Material

Supplemental data

ICMJE disclosure forms

Supporting data values

## Version 1 08/07/2025

In-Press Preview

## Version 2 09/23/2025

Electronic publication
