# Supplementary material for: Longitudinal single-cell analysis of glucagon-like peptide-2 treatment in patients with short bowel syndrome
Source: JCI Insight. 2025 Aug 7;10(18):e194497. doi: 10.1172/jci.insight.194497 (PMC12487850; doi:10.1172/jci.insight.194497)
Supplement: Supplemental data [file jciinsight-10-194497-s057.pdf]

Methods

Sex as a biological variable

Our study involved five Japanese male participants. Race and ethnicity were assessed by the investigators based on medical records. No other demographic groups were represented due to the limited availability of eligible patients.

Patients

Five patients were recruited from Keio university Hospital. All patients underwent extensive resection of the small intestine because of intestinal volvulus or vascular ischemia, with residual small intestine of 0-40 cm and have colon in continuity. All patients have no clinical suspicion of active inflammatory bowel disease or intestinal motility dysfunction. The characteristics of these patients are shown in Table 1.

Study protocol

All patients were admitted to the hospital before, 6 and 12 months after the start of teduglutide administration, and underwent blood tests to evaluate electrolytes and nutritional status and balloon-assisted enteroscopy to ascertain the condition of the

intestinal mucosa and obtain biopsy and fecal samples. Blood tests were performed in the fasting state. The biopsy was performed approximately 10 cm proximal to the anastomosis between the small intestine and the colon, with a portion submitted for histological evaluation and the remainder for single cell analysis. Estimated residual small bowel and colon lengths were determined by reviewing operative reports and endoscopic findings. The fecal samples were collected from the wash solution during enteroscopy. During each inpatient period, weight, urine and fecal output were measured for 3-5 days and averages were calculated.

Teduglutide was injected subcutaneously into the abdomen or thigh. Three patients received 0.05 mg/kg/day and the other two patients with moderate or greater renal dysfunction received 0.025 mg/kg/day once daily.

Histological analysis

Tissue samples for histological analysis were obtained from endoscopic biopsies. They are fixed with 10% buffered formalin and stained with hematoxylin-eosin (HE). They were digitized using the NanoZoomer 2.2 Digital Pathology system (Hamamatsu Photonics) and analyzed using NDP.view2 software. Measurements and evaluations were conducted by two experts: a pediatric surgeon and a pathologist. The pathologist

performed the evaluation with access only to the patient's diagnosis, without any clinical course or examination results. The parameters evaluated were villus height, villus width, crypt depth, total length of villus, villus surface area, the number of intestinal epithelial (IE) cells per villus and mitotic index. Crypt depth was measured from the base to the transition region between the crypts and the villi upward (1). Villus height was measured as the distance between the base and the apex of each villus. Villus width was assessed in the transitional zone between the crypts and the villi, including the two epithelial monolayers and the lamina propria (2). Total length of villus was calculated by adding villus height and crypt depth. Villus surface area was calculated using the formula $(2\pi)(VW/2)(VL)$, where VW = villus width, and VL = villus length (3) (4). The number of IE cells was counted per villus. These counts were performed in both sides of each villus monolayer (5). For measurement of the mitotic index in the crypt, ten crypts were randomly selected in each sample, and the cell mitosis number was counted within one transverse section (6). Villus height, villus width and crypt depth were measured as the mean of 1-10 well oriented crypts and villi.

16S ribosomal RNA (16S rRNA) gene analysis

Fresh small bowel stool samples were collected, frozen in 20% glycerol and stored at -

80 °C until processing. DNA isolation from stool samples and sequencing for the 16S rRNA gene analysis were outsourced to the Bioengineering Laboratory. Co., Ltd. Genomic DNA was extracted from the stool samples. The stool samples stored at -80 °C was thawed, and Lysis Solution F (NIPPON GENE) was added; the mixture was crushed at 1,500 rpm for 2 min using a Shake Master Neo (BIO MEDICAL SCIENCE). After incubation at 65 °C for 10 min, centrifugation was performed at 12,000 x g for 2 min, and the supernatant was separated. DNA was purified from the aliquoted solution using a Lab-Aid®824s DNA Extraction kit (Zeesan Biotech）. Libraries were prepared using a two-step tailed polymerase chain reaction (16S rRNA V3-V4) and sequenced at 2 × 300 bp using the MiSeq system and MiSeq Reagent Kit v3 (Illumina). Cutadapt (version 4.6) is trimming primer sequences (Forward: CCTACGGGNGGCWGCAG, Reverse: GACTACHVGGGTATCTAATCC) from sequencing reads (7). Quality filtering, merge read pairs, polymerase chain reaction (PCR) chimera removal and infers exact Amplicon Sequence Variants (ASVs) was used DADA2 (version 1.30.0) (8). Barrnap (version 0.9) was used to remove Amplicon Sequence Variants (ASVs) other than bacteria (https://github.com/tseemann/barrnap). The representative sequences and ASVs tables were obtained after removing the chimeric and noise sequences using the Quantitative Insights Into Microbial Ecology (QIIME) 2 plugin (version 2023.7.0) (9-11).

Phylogenetic inference was performed by comparing the Silva 138 99% operational taxonomic units (OTUs) full-length sequences with the representative sequences obtained using the feature-classifier plugin (12). Three samples were removed because the taxonomic string contained any of mitochondria, chloroplast, archaea and unclassified. Diversity analysis was performed using QIIME2. Diversity calculations are based on sub-sampled data rarefied to 19,009 count. Alpha diversity was compared using the observed OTUs; beta diversity was estimated based on Bray-Curtis dissimilarity, weighted Unique Fraction metric (UniFrac) and unweighted UniFrac distances, and visualized using principal coordinate analysis (PCoA). Statistical analysis of beta diversity was performed using permutational multivariate analysis of variance (PERMANOVA), while alpha diversity was analyzed using one-way ANOVA followed by Tukey's multiple comparisons post-hoc test. Bar plots were created using microshades (version 1.12) (13). Differential microbiota abundance was analyzed by the analysis of compositions of microbiomes with bias correction 2 (ANCOM-BC2) (14, 15) using the qiime2 command. To predict the function of microbiomes, we used Phylogenetic Investigation of Communities by Reconstruction of Unobserved States (PICRUSt2). Pathway and enzyme commission (EC) abundance predictions were performed using QIIME 2 plugins via the following commands: qiime picrust2 full-pipeline, qiime feature-table summarize, and

qiime diversity core-metrics. Sampling depth (–p-sampling-depth) was set to the minimum number of remaining read counts among samples (16).

Isolation of intestinal epithelial (IE) cells and lamina propria (LP) cells as single cells from human samples

The intestinal tissue was washed with Ca_2^+, Mg_2^+-free Hank's balanced salt solution (HBSS) (Nacalai Tesque, 17460-15) to remove fecal content. After washing, the intestinal tissue was incubated with HBSS containing 1 mM dithiothreitol (Invitrogen, 15508-013) and 1 mM EDTA (Nacalai Tesque, 06894-85) for 15 min at 37°C. The supernatant of the solution was harvested for IE cells by passing through a 100-μm cell strainer into a 50-mL Falcon tube. At the same time, the tissues without the IE cells were washed with HBSS and dissolved into solutions by incubation with HBSS containing 2.5% fetal bovine serum (FBS) (Thermo Fisher Scientific, 10270-106) and penicillin/streptomycin (Nacalai Tesque, 26253-84), 1.0 mg/mL collagenase Type3 (Worthington, 032-22364), and 0.1 mg/mL DNase (Sigma-Aldrich, DN25) for 60 min at 37 °C. The dissolved solution and digested tissues were homogenized by vigorous shaking and passed through a 100-mm cell strainer into a 50-mL Falcon tube. The solution was centrifuged at 1,700 rpm for 5 min at 4°C , the supernatant was removed, and resuspended in RPMI-1640

(Nacalai Tesque, 00-5523-02) containing 10% FBS and penicillin/streptomycin (Nacalai Tesque, 26253-84) (17, 18).

Single-cell RNA sequencing (ScRNA-seq)

Isolated single cell suspensions of IE and LP cells were pooled and incubated with a Fixable Viability Dye eFluor (eBioscience, 65-0865-14) for 15 min at 4 °C. Live cells were sorted using BD FACS Aria-II (BD Bioscience) and loaded into Chromium Controller (10X Genomics). RNA-seq libraries were prepared using Chromium single-cell 5' gene expression library preparation (human) according to the manufacturer's instructions (10X Genomics). The libraries were then sequenced using a DNBSEQ (MGI Tech). Sequence reads from all samples were processed by Cell Ranger (10X Genomics). Seurat (v 5.1.0) (19) was used to aggregate and analyze the processed data following the official vignettes (https://satijalab.org/seurat/articles/sctransform_vignette.html). Specifically, Principal Component Analysis (PCA) analysis was performed to identify clusters, and 20 gene clusters (0–20) were projected on Uniform Manifold Approximation and Projection (UMAP) space with the normalized gene expression. Gene ontogeny analysis was performed using clusterProfiler (v4.12.6) (20).

Cell–cell communication analysis using CellChat

To investigate intercellular communication networks, we applied the CellChat R package (v1.6.1) (21) to single-cell transcriptomic data. Raw expression matrices were processed using the Seurat pipeline, and cell-type annotations were integrated into the CellChat object for downstream analysis. For each time point (0M and 12M), a separate CellChat object was initialized and the human ligand–receptor interaction database provided in CellChatDB.human was used. We subsetted the database to include signaling interactions relevant to the secreted signaling class. Pathway names such as TGFβ, CCL, and GALECTIN refer to CellChat-defined signaling groups and may represent multiple genes or ligand families rather than individual gene symbols.

Statistics

All values are shown as mean ± SD. Statistical analyses were performed using GraphPad Prism 9 (GraphPad Software). To compare clinical data between two groups, statistical differences were evaluated using the one-tailed paired Student's t-test. For data that were not normally distributed, log transformation was applied (using ln(x) for values greater than zero) to achieve normality, followed by the one-tailed paired Student's t-test on the transformed data. Histological analysis was evaluated using the Mann-Whitney U test.

Microbiome analyses were assessed using PERMANOVA for beta diversity, the Mann-Whitney U test for alpha diversity, ANCOM-BC2 for differential microbiota abundance and the Friedman test for microbial functional prediction. For scRNA-seq data analysis, the two-tailed paired Student's t-test was used to compare cluster proportions. DEGs were determined by Wilcoxon rank-sum test. GO analysis and CompareCluster analysis were performed using Over-Representation Analysis (ORA) based on a hypergeometric test. For CompareCluster analysis, multiple testing correction was performed using the Benjamini-Hochberg (BH) method to control the false discovery rate (FDR). To compare gene expression between clusters, the Wilcoxon rank-sum test was used.

A *P* value less than 0.05 were considered statistically significant.

Study approval

All experiments were approved (20221003) by the Institutional Review Board of Keio University School of Medicine, and written informed consent was obtained from all patients in accordance with the Declaration of Helsinki.

Data availability

Underlying data points are provided in the Supporting Data Values document. All data supporting the findings of this study are available from the corresponding author upon reasonable request. Single-cell RNA-sequencing (scRNA-seq) data have been deposited in the Gene Expression Omnibus (GEO) under accession number GSE294205. The 16S rRNA gene sequencing data have been deposited in the DDBJ Sequence Read Archive under accession number PRJDB20353.

Reference

1. Aptekmann KP, Baraldi Arton SM, Stefanini MA, and Orsi MA. Morphometric analysis of the intestine of domestic quails (Coturnix coturnix japonica) treated with different levels of dietary calcium. *Anat Histol Embryol.* 2001;30(5):277-80.
2. Pereira ESA, Soares JRA, Mattos EBA, Josetti C, Guimaraes IM, Campos SMN, et al. A histomorphometric classification system for normal and inflamed mouse duodenum-Quali-quantitative approach. *Int J Exp Pathol.* 2018;99(4):189-98.
3. Sakamoto K, Hirose H, Onizuka A, Hayashi M, Futamura N, Kawamura Y, et al. Quantitative study of changes in intestinal morphology and mucus gel on total parenteral nutrition in rats. *J Surg Res.* 2000;94(2):99-106.
4. Zhang W, Frankel WL, Adamson WT, Roth JA, Mantell MP, Bain A, et al. Insulin-like growth factor-I improves mucosal structure and function in transplanted rat small intestine. *Transplantation.* 1995;59(5):755-61.
5. Hasan M, and Ferguson A. Measurements of intestinal villi non-specific and ulcer-associated duodenitis-correlation between area of microdissected villus and villus epithelial cell count. *J Clin Pathol.* 1981;34(10):1181-6.
6. Sung D, Iuga AC, Kato T, Martinez M, Remotti HE, and Lagana SM. Crypt apoptotic body counts in normal ileal biopsies overlap with graft-versus-host disease and acute cellular rejection of small bowel allografts. *Hum Pathol.* 2016;56:89-92.
7. Kechin A, Boyarskikh U, Kel A, and Filipenko M. cutPrimers: A New Tool for Accurate Cutting of Primers from Reads of Targeted Next Generation Sequencing. *J Comput Biol.*

2017;24(11):1138-43.

8. Callahan BJ, McMurdie PJ, Rosen MJ, Han AW, Johnson AJ, and Holmes SP. DADA2: High-resolution sample inference from Illumina amplicon data. *Nat Methods.* 2016;13(7):581-3.

9. Bolyen E, Rideout JR, Dillon MR, Bokulich NA, Abnet CC, Al-Ghalith GA, et al. Reproducible, interactive, scalable and extensible microbiome data science using QIIME 2. *Nat Biotechnol.* 2019;37(8):852-7.

10. Miyamoto K, Sujino T, Harada Y, Ashida H, Yoshimatsu Y, Yonemoto Y, et al. The gut microbiota-induced kynurenic acid recruits GPR35-positive macrophages to promote experimental encephalitis. *Cell Rep.* 2023;42(8):113005.

11. Kubosawa Y, Sujino T, Miyamoto K, Kayashima A, Minezaki D, Morioka K, et al. Distinctive duodenal microbiomes and bile acid profiles in duodenal tumor patients revealed by prospective observational study. *Sci Rep.* 2024;14(1):18705.

12. Quast C, Pruesse E, Yilmaz P, Gerken J, Schweer T, Yarza P, et al. The SILVA ribosomal RNA gene database project: improved data processing and web-based tools. *Nucleic Acids Res.* 2013;41(Database issue):D590-6.

13. Dahl EM, Neer E, Bowie KR, Leung ET, and Karstens L. microshades: An R Package for Improving Color Accessibility and Organization of Microbiome Data. *Microbiol Resour Announc.* 2022;11(11):e0079522.

14. Lin H, and Peddada SD. Multigroup analysis of compositions of microbiomes with covariate adjustments and repeated measures. *Nat Methods.* 2024;21(1):83-91.

15. Mandal S, Van Treuren W, White RA, Eggesbø M, Knight R, and Peddada SD. Analysis of composition of microbiomes: a novel method for studying microbial composition. *Microb Ecol Health Dis.* 2015;26:27663.

16. Douglas GM, Maffei VJ, Zaneveld JR, Yurgel SN, Brown JR, Taylor CM, et al. PICRUSt2 for prediction of metagenome functions. *Nat Biotechnol.* 2020;38(6):685-8.

17. Tanemoto S, Sujino T, Miyamoto K, Moody J, Yoshimatsu Y, Ando Y, et al. Single-cell transcriptomics of human gut T cells identifies cytotoxic CD4(+)CD8A(+) T cells related to mouse CD4 cytotoxic T cells. *Front Immunol.* 2022;13:977117.

18. Yoshimatsu Y, Sujino T, Miyamoto K, Harada Y, Tanemoto S, Ono K, et al. Aryl hydrocarbon receptor signals in epithelial cells govern the recruitment and location of Helios(+) Tregs in the gut. *Cell Rep.* 2022;39(6):110773.

19. Hao Y, Stuart T, Kowalski MH, Choudhary S, Hoffman P, Hartman A, et al. Dictionary learning for integrative, multimodal and scalable single-cell analysis. *Nat Biotechnol.* 2024;42(2):293-304.

20. Yu G, Wang LG, Han Y, and He QY. clusterProfiler: an R package for comparing biological

themes among gene clusters. *Omics.* 2012;16(5):284-7.

21. Jin S, Guerrero-Juarez CF, Zhang L, Chang I, Ramos R, Kuan CH, et al. Inference and analysis of cell-cell communication using CellChat. *Nat Commun.* 2021;12(1):1088.

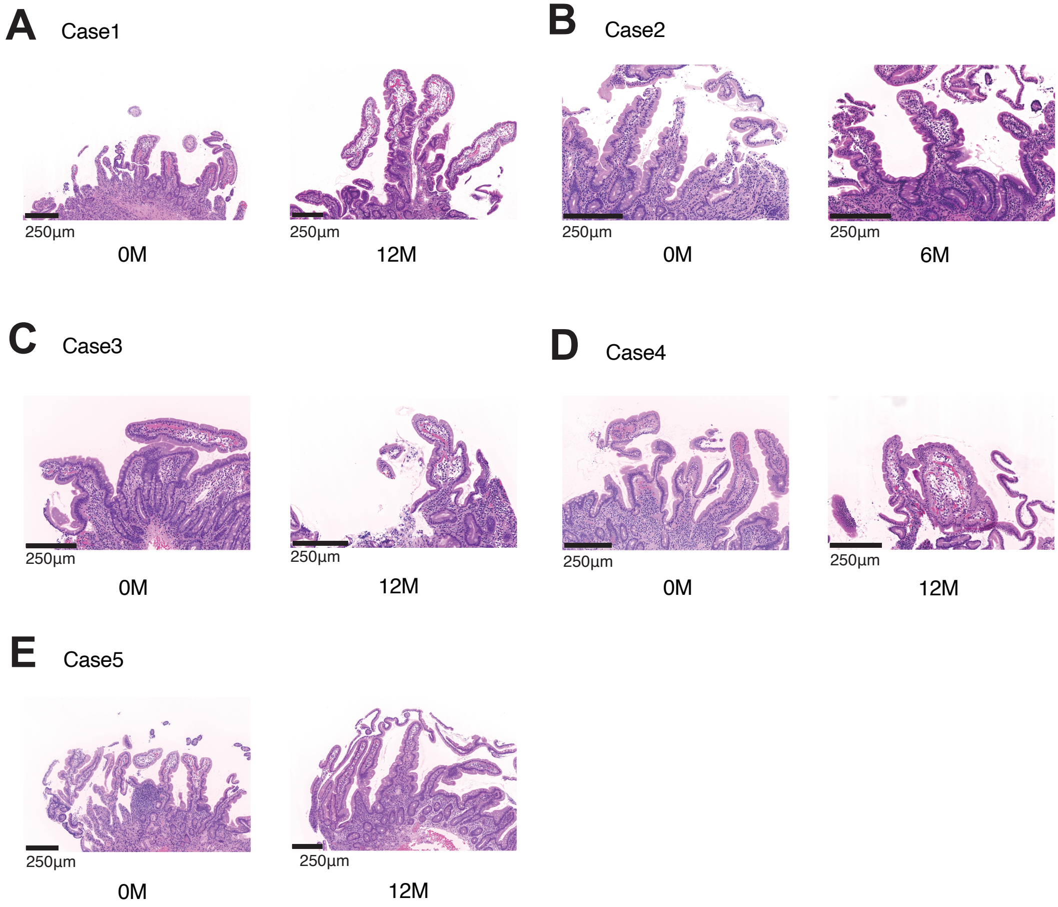

Supplemental Figure 1 (related to Figure 1). Histological analysis of intestinal villi in patients
(**A**-**E**) HE-stained specimens of cases 1–5, with the scale bar shown at the bottom left.

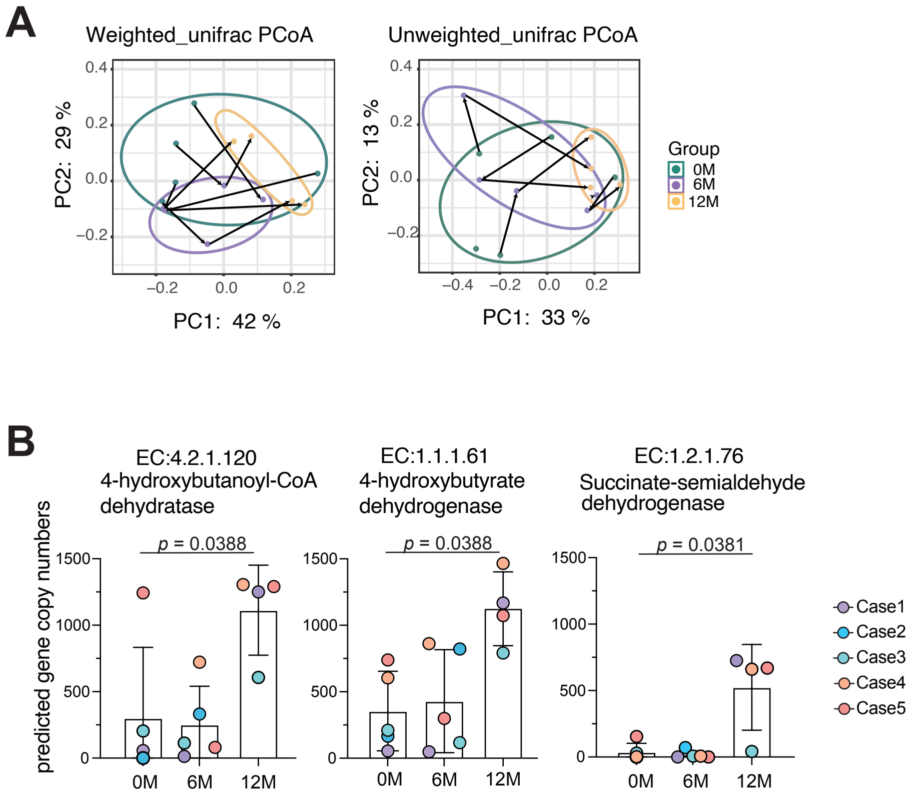

Supplemental Figure 2 (related to Figure 2). Microbial Diversity and EC abundance related to the succinate–butanoate pathway
(**A**) β-Diversity Analysis: Principal Coordinates Analysis (PCoA) based on weighted Unique Fraction metric (UniFrac) (left panel) and unweighted UniFrac (right panel) distances to assess gut microbiota differences among samples. (**B**) Enzyme commission (EC) abundance: Predicted gene copy numbers of EC:4.2.1.120 (left panel), EC:1.1.1.61 (middle panel) and EC:1.2.1.76 (right panel) which showed significant temporal differences by Friedman test and exhibited increased abundance at 12M compared to 0M and/or 6M. Each point represents the predicted gene copy number across the three time points. Statistical analyses included permutational multivariate ANOVA for (**A**) and Friedman test for (**B**).

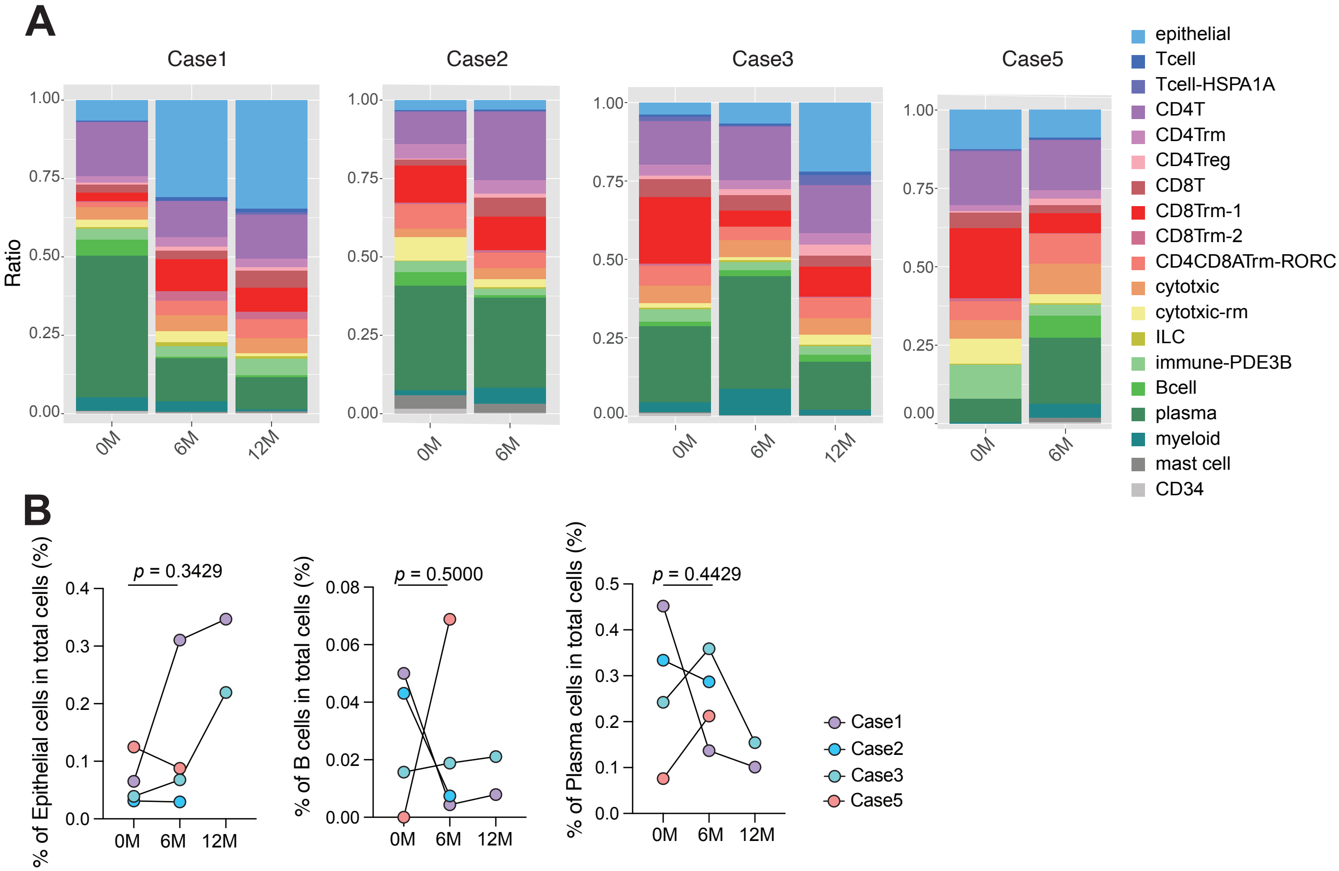

Supplemental Figure 3 (related to Figure 3). Proportion of each cell cluster in individual patients at 0M, 6M, and 12M
(**A**) Cluster proportions in individual patients at 0M, 6M, and 12M. (**B**) The percentage of epithelial cells, Bcells and plasma cells at 0M, 6M, and 12M. The two-tailed paired Student's t-test was used to compare cluster proportions (**B**).

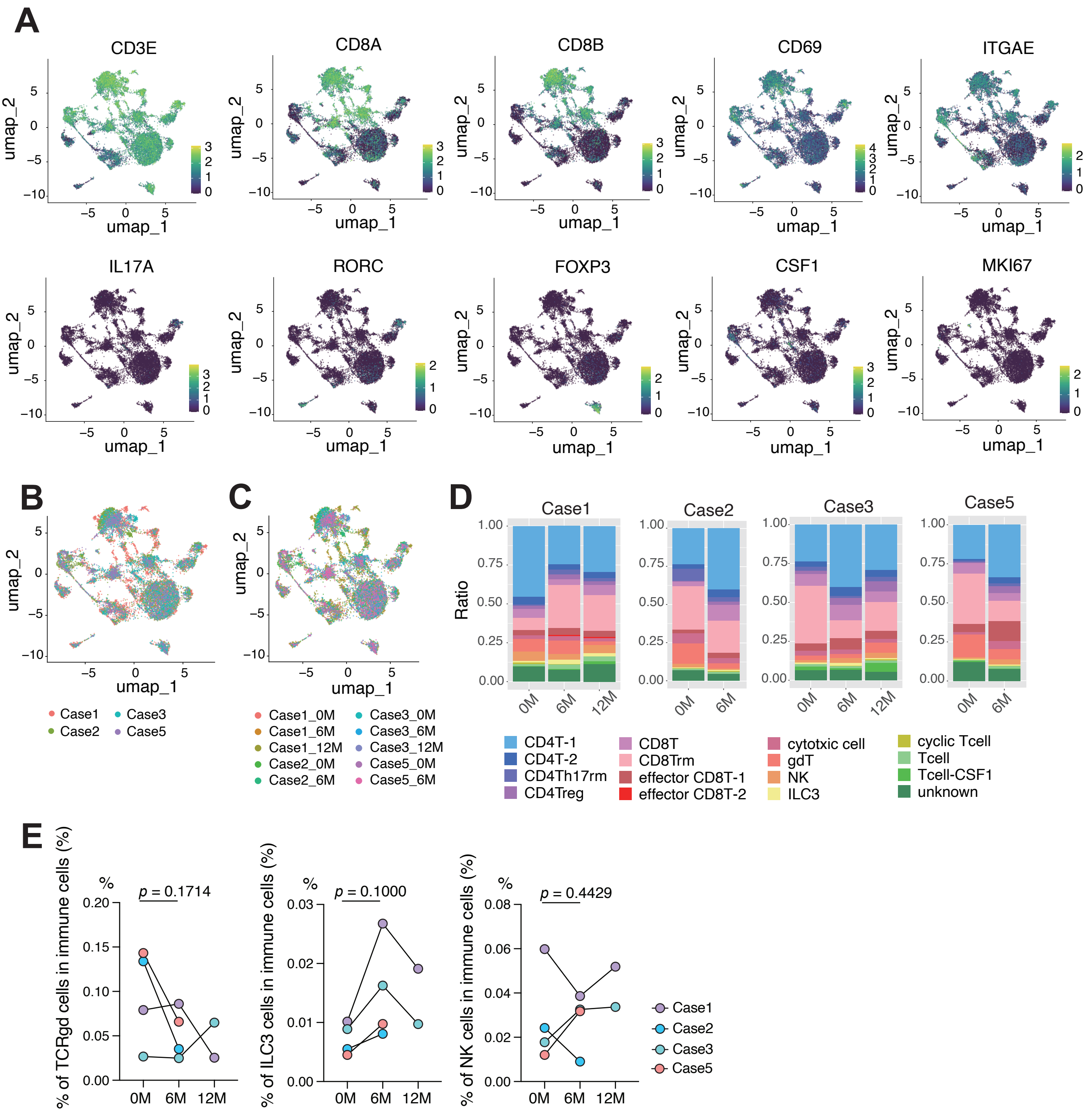

Supplemental Figure 4 (related to Figure 4). Gene Expression Patterns of Immune cells

(**A**) Expression of immune markers (*CD3E*, *CD8A*, *CD8B*, *CD69*, *ITGAE*, *IL17A*, *RORC*, *FOXP3*, *CSF1*, *MKI67*) in Uniform Manifold Approximation and Projection (UMAP). (**B**) UMAP colored by patient case. (**C**) UMAP colored by case and time point. (**D**) Cluster proportions in individual patients at 0M, 6M, and 12M. (**E**) The percentage of T-cell receptor gamma-delta (TCRγδ) cells, group 3 innate lymphoid cells (ILC3s) and NK cells at 0M, 6M, and 12M. The two-tailed paired Student's t-test was used to compare cluster proportions (**E**).

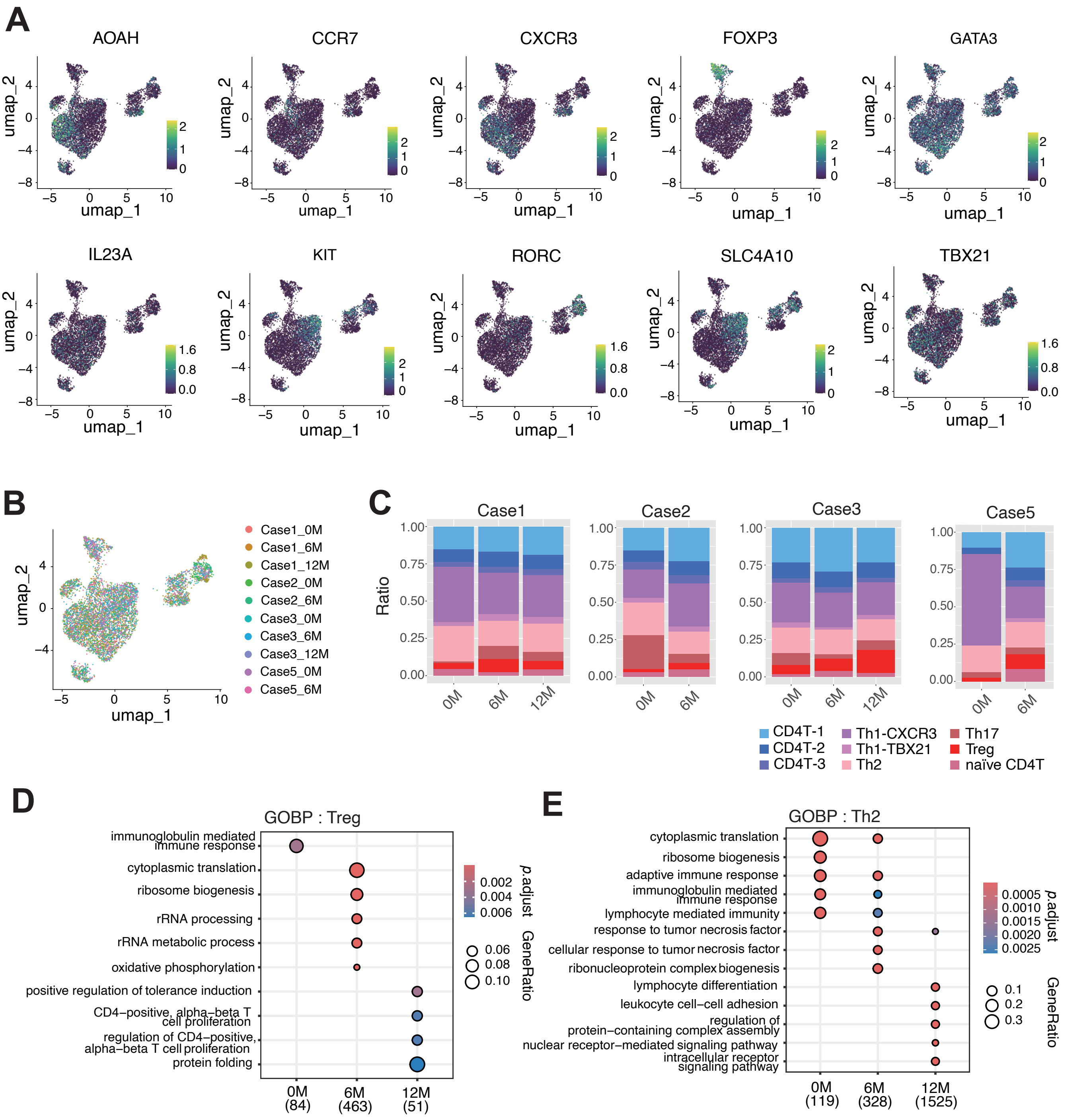

Supplemental Figure 5 (related to Figure 5). Gene Expression in CD4+ T Cells and GO enrichment analyses for Treg and Th2 cell populations
(**A**) UMAP visualization of CD4+ T cells showing the expression of *AOAH*, *CCR7*, *CXCR3*, *FOXP3*, *GATA3*, *IL23A*, *KIT*, *RORC*, *SLC4A10*, and *TBX21*. (**B**) UMAP plots of CD4+ T cells, colored by patient case and time point. (**C**) Proportions of CD4+ T cell clusters in each patient at three time points: 0M, 6M, and 12M. (**D** and **E**) Gene Ontology (GO) Biological Process (BP) enrichment analysis of differentially expressed genes (DEGs) for Treg (**D**) and Th2 (**E**) clusters across 0M, 6M, and 12M using compareCluster. Significantly enriched GO terms (y-axis) with $p < 0.01$ and $q < 0.05$ are visualized as dot plots. Numbers below the column name represent the number of genes related to GOBP for each cluster. Dot size represents the gene ratio (proportion of DEGs), and color indicates the adjusted *p*-value. CompareCluster analysis was performed using Over-Representation Analysis (ORA) based on a hypergeometric test. Multiple testing correction was applied using the Benjamini-Hochberg (BH) method to control the false discovery rate (FDR) (**D** and **E**).

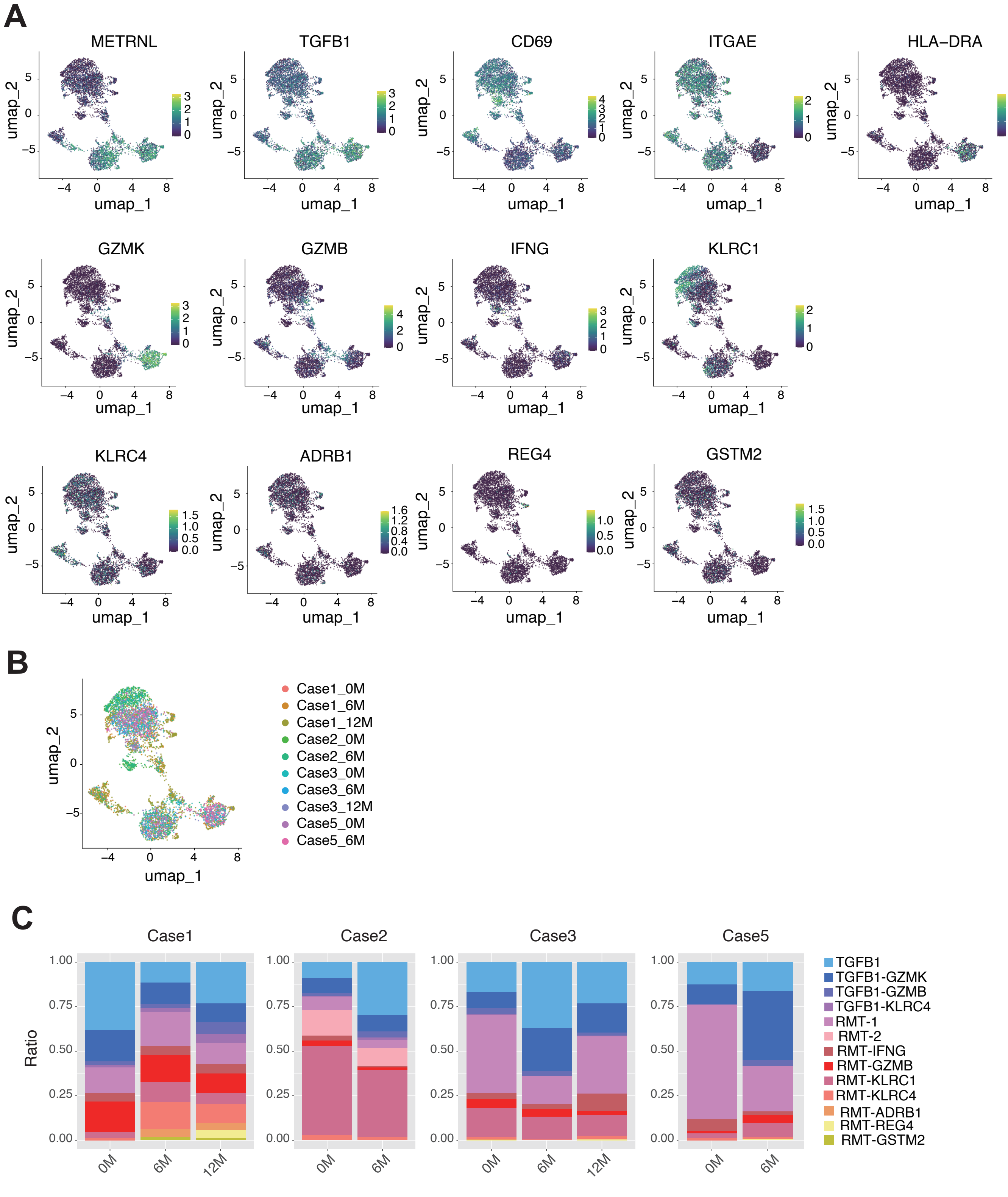

Supplemental Figure 6 (related to Figure 6). Gene Expression in CD8+ T Cells from Four Patients with SBS
(**A**) UMAP visualization of CD8+ T cells showing the expression of *METRNL*, *TGFB1*, *CD69*, *ITGAE*, *HLA-DRA*, *GZMK*, *GZMB*, *IFNG*, *KLRC1*, *KLRC4*, *ADRB1*, *REG4*, and *GSTM2*. (**B**) UMAP plots of CD8+ T cells, colored according to patient cases and time points. (**C**) Proportions of CD8+ T cell clusters in each patient at three time points: 0M, 6M, and 12M.

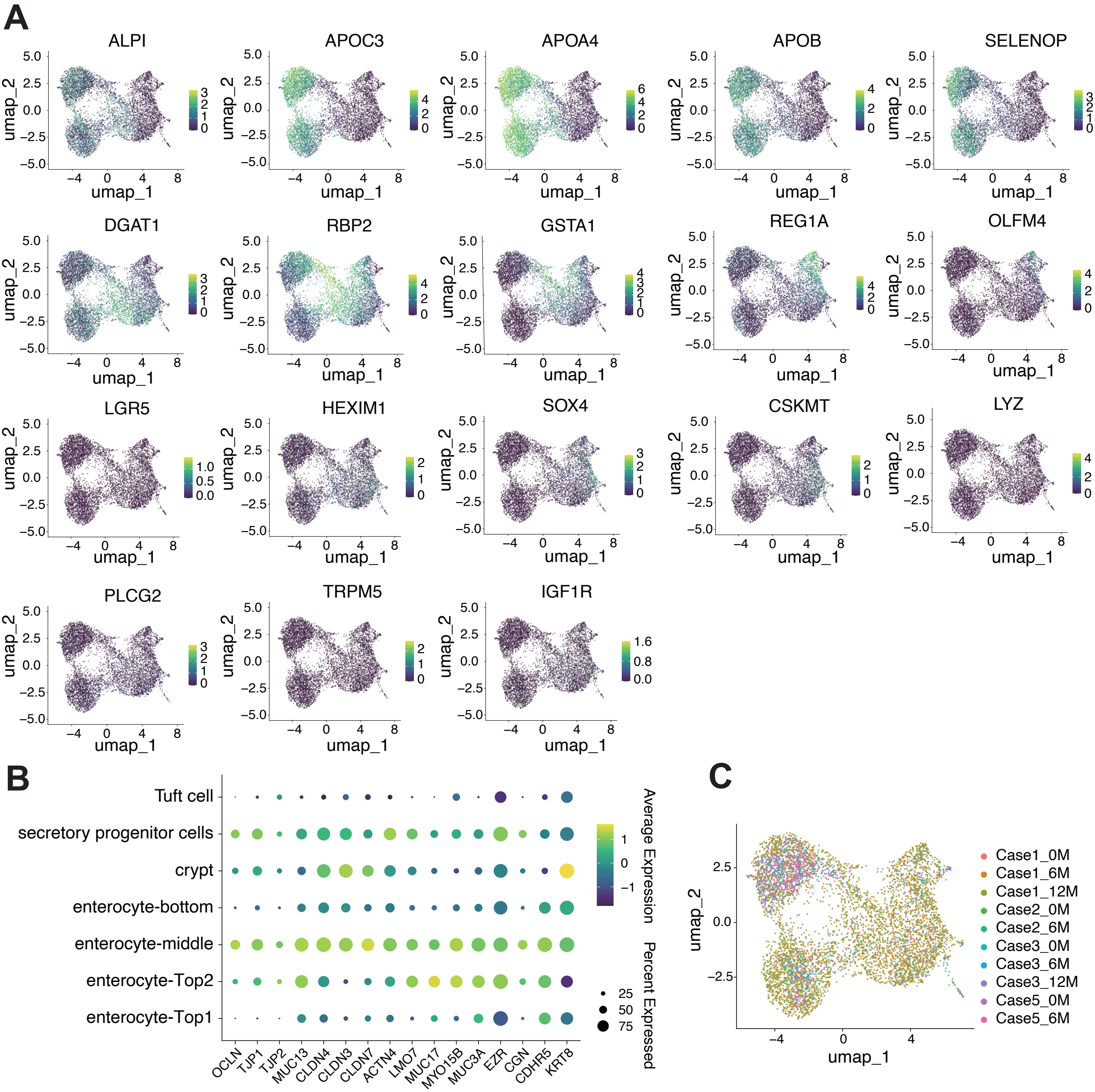

Supplemental Figure 7 (related Figure 7, 8). Gene Expression Patterns of Epithelial Cells.
(**A**) UMAP visualization of epithelial cells, showing the expression of *ALPI*, *APOC3*, *APOA4*, *APOB*, *SELENOP*, *DGAT1*, *RBP2*, *GSTA1*, *REG1A*, *OLFM4*, *LGR5*, *HEXIM1*, *SOX4*, *CSKMT*, *LYZ*, *PLCG2*, *TRPM5*, and *IGF1R*. (**B**) Expression patterns of barrier function-related genes across epithelial cell clusters. Each dot represents a gene, with color saturation indicating scaled expression levels and dot size representing the percentage of cells expressing the gene. (**C**) UMAP plot of epithelial cells, colored by patient cases and time points.

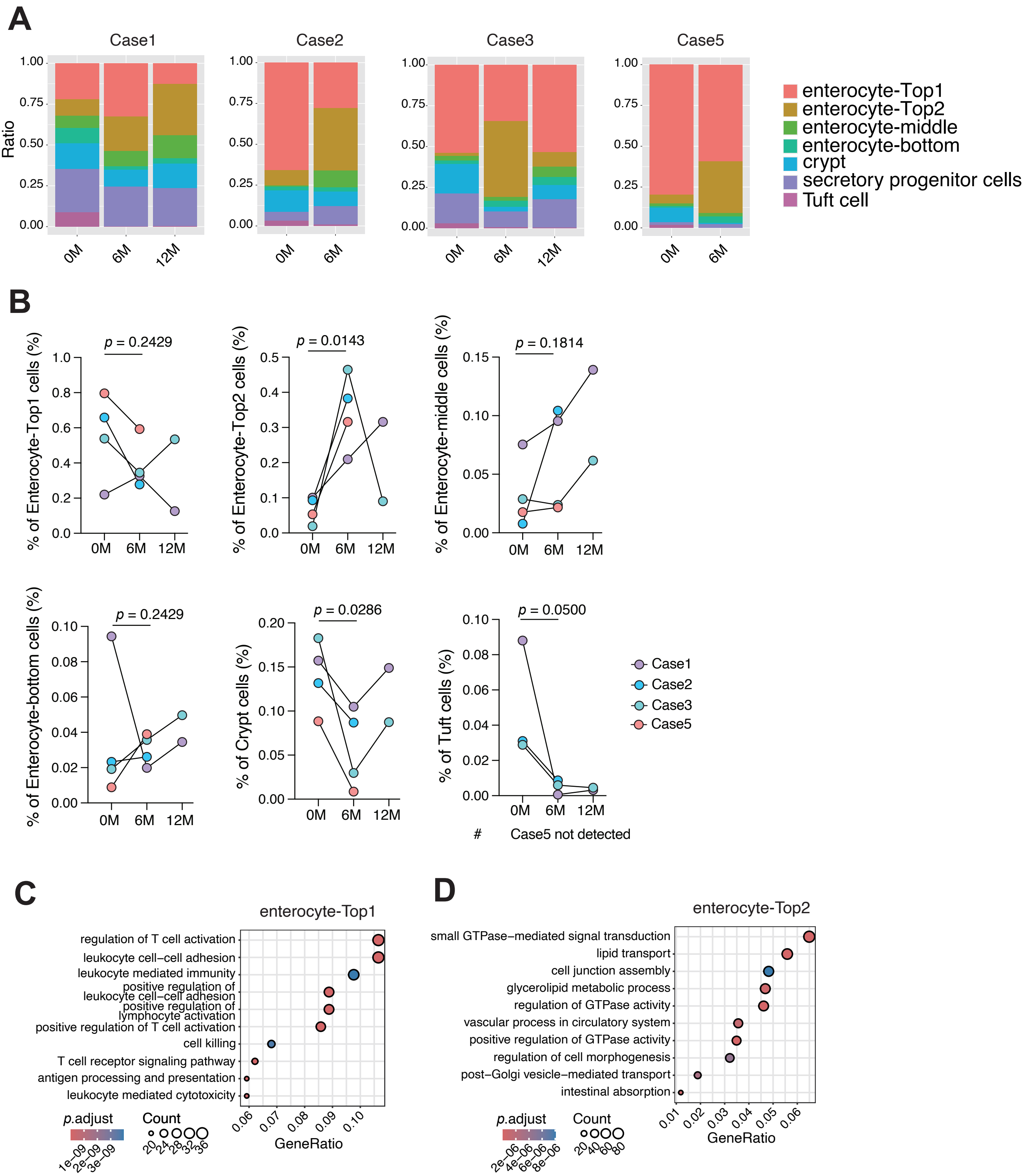

Supplemental Figure 8 (related to Figure 7, 8). Gene Expression Patterns and Functional Analysis of Epithelial Cells
(**A**) Proportions of epithelial cell clusters for each patient at 0M, 6M, and 12M. (**B**) The percentage of enterocyte-Top1 cells, enterocyte-Top2 cells, enterocyte-middle cells, enterocyte-bottom cells, crypt cells and tuft cells at 0M, 6M, and 12M. (**C** and **D**) GO pathway enrichment analysis for the "enterocyte-Top1" (**C**) and "enterocyte-Top2" (**D**) clusters, highlighting significantly enriched biological processes. Genes with Log2FC > 0.58 were analyzed. Dot plots show significantly enriched GO terms ($p < 0.01$, $q < 0.05$), where dot size represents the number of DEGs and color indicates the adjusted *p*-value. The two-tailed paired Student's t-test was used to compare cluster proportions (**B**).GO analysis was performed using ORA based on the hypergeometric test. (**C** and **D**).

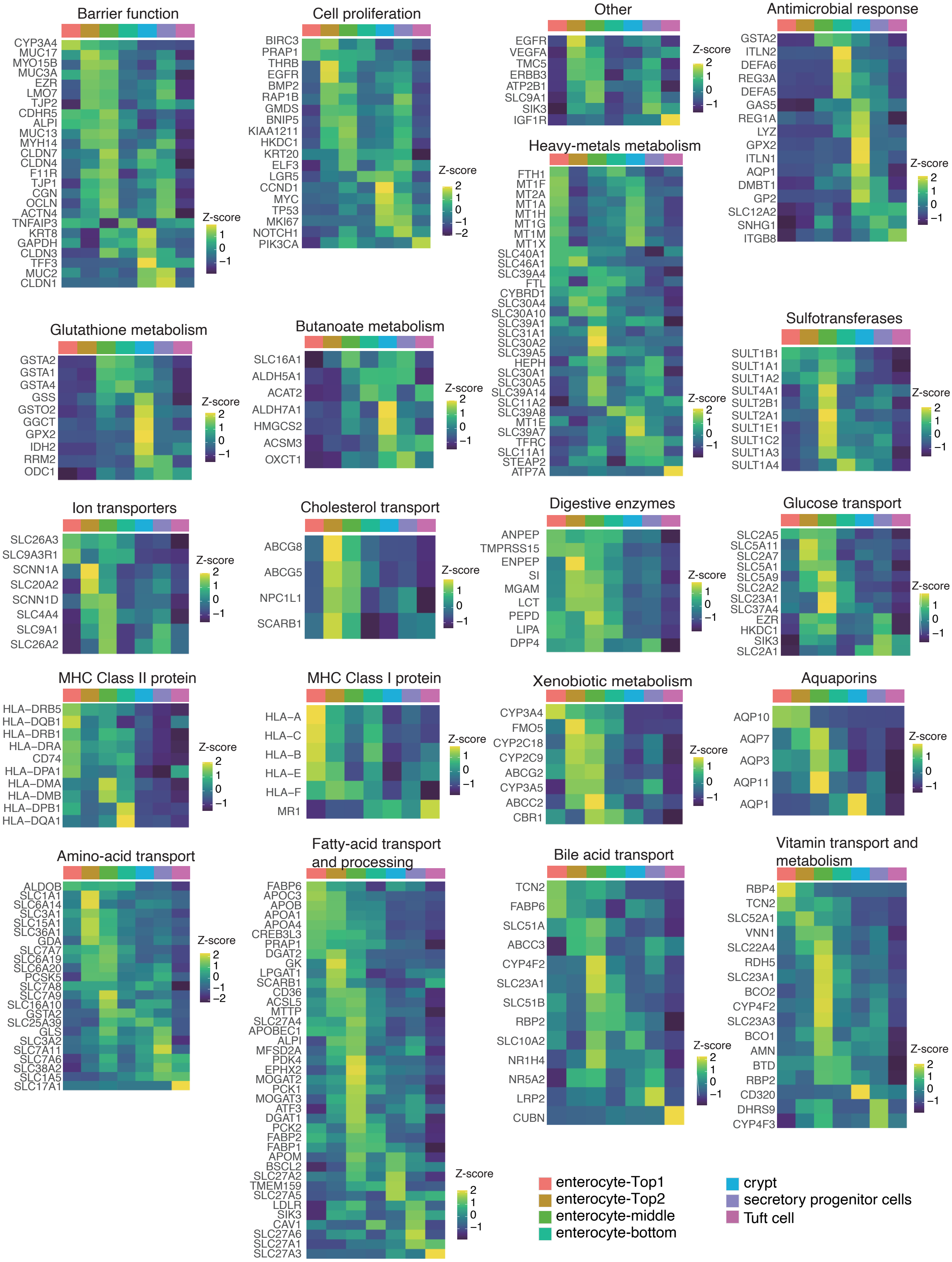

Supplemental Figure 9 (related to Figure 7). Gene Expression Patterns and Pattern Analysis of Epithelial Cells
Heatmap showing Z-score normalized gene expression of key intestinal mucosal pathways (same as Figure 7E) across clusters. Columns represent clusters (indicated by color and label), and rows represent genes categorized by pathway. To compare gene expression between clusters, the Wilcoxon rank-sum test was used.

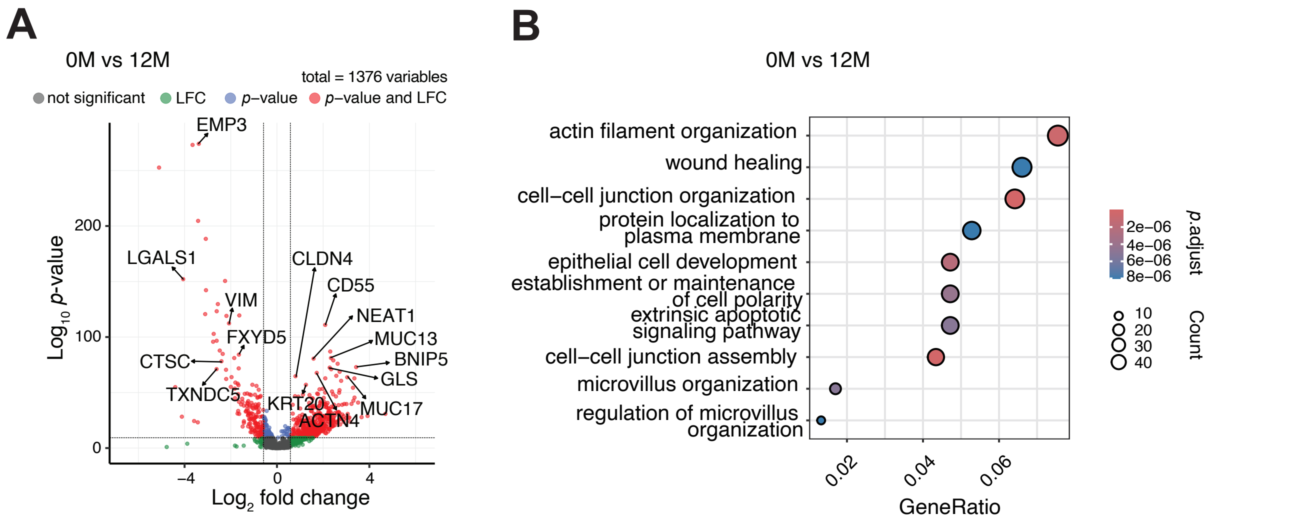

Supplemental Figure 10 (related to Figure 7, 8). Gene Expression Patterns of Epithelial Cells at 12M
(**A**) Volcano plot showing DEGs in epithelial cells at 0M vs 12M. The x-axis represents log2 fold change (LFC), and the y-axis represents -log10 (*p*-value). Vertical dashed lines indicate a threshold of |LFC| > 0.58, and the horizontal dashed line indicates a *p*-value cutoff of 5×10^{-10}. (**B**) GO pathway enrichment analysis of DEGs between 0M and 12M. Genes with LFC > 0.58 that were upregulated at 12M were analyzed. Dot plots display significantly enriched biological processes ($p < 0.01$, $q < 0.05$), with dot size indicating gene count and color representing the adjusted *p*-value. DEGs were determined by Wilcoxon rank-sum test (**A**). GO analysis was performed using ORA based on the hypergeometric test (**B**).

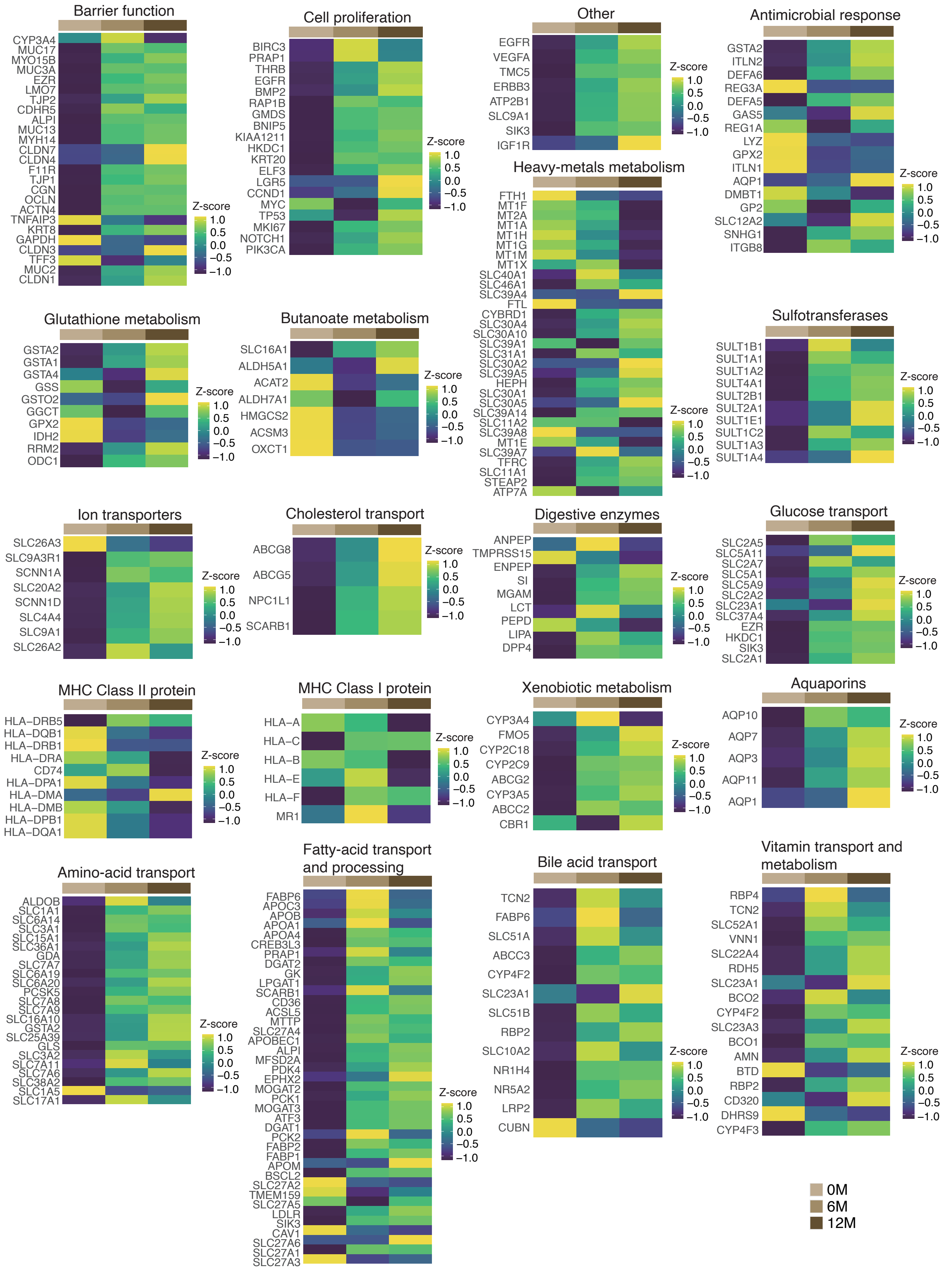

Supplemental Figure 11 (related to Figure 8). Gene Expression Patterns and Pattern Analysis of Epithelial Cells

Heatmap showing Z-score normalized gene expression of key intestinal mucosal pathways (same as Figure 8E) at 0M, 6M, and 12M. Columns represent the time points (indicated by color and label), and rows represent genes categorized by pathway. To compare gene expression between clusters, the Wilcoxon rank-sum test was used.

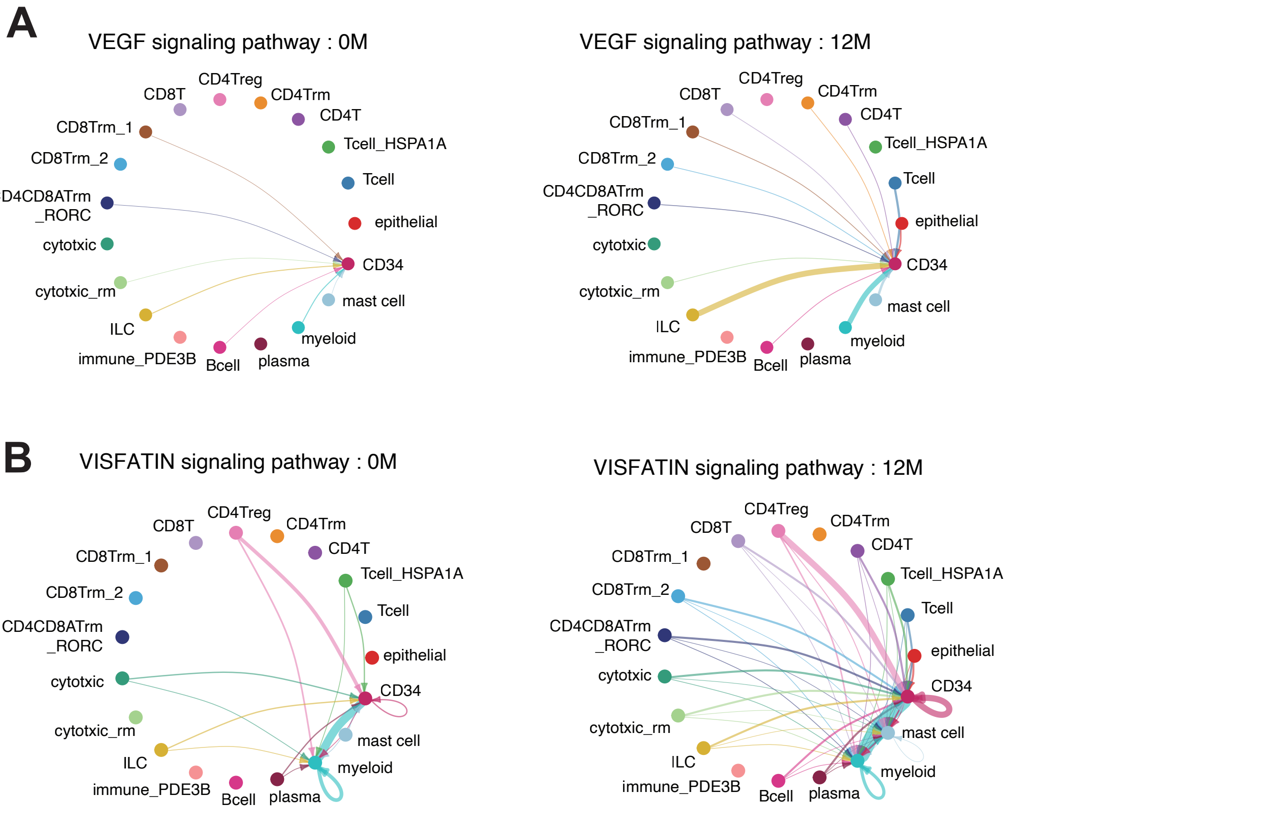

Supplemental Figure 12 (related to Figure 10, 11). Signaling networks between cell clusters.
(**A** and **B**) Circle plots showing intercellular signaling networks. The VEGF (**A**) and VISFATIN (**B**) signaling pathways were upregulated at 12M compared to 0M. Arrow colors indicate the source (sender) clusters of the signaling, and arrow widths represent the relative signaling strength.

Table 1. Clinical data sheet of the patients

Case	Age (yr)	Sex	Cause of SBS	Duodenum (cm)	Small bowel (cm)	Colon
1	59	Male	Acute mesenteric ischemia	All	30	Below the transverse colon
2	33	Male	Intestinal volvulus	40	0	Below the transverse colon
3	39	Male	Small intestinal leiomyoma	35	0	Below the cecum
4	37	Male	Aortic dissection	All	10	Below the sigmoid colon
5	18	Male	Intestinal volvulus	All	40	Below the ascending colon

Short bowel syndrome (SBS).

Table 2. Histological analysis of each patient

Sample	Villus height (μm)	Villus width (μm)	Crypt depth (μm)	Total length of villus (μm)	Villus surface area (mm^2)	Number of epithelia cells/ villus	Mitotic index
Case1							
Baseline	250±27.3	91.2±8.0	87.6±8.2	338	0.0717	129±14.4	1/10
6month	603±90.0	155±21.9	312±32.8	915	0.293	269±48.5	5/10
12month	592±58.4	160±21.3	408±21.1	1000	0.298	240±33.7	5/10
Case2							
Baseline	416±35.7	119±8.4	172±29.9	588	0.156	181±39.5	0/10
6month	476±18.8	115±4.2	206±24.5	682	0.172	167±26.9	0/10
12month	N/A[A]	N/A[A]	N/A[A]	N/A[A]	N/A[A]	N/A[A]	N/A[A]
Case3							
Baseline	334±40.1	168±18.5	267±11.0	601	0.176	132±24.2	1/10
6month	332±42.6	107±13.0	180	512	0.112	121±24.3	3/10
12month	465±79.3	127±19.5	356±10.3	820	0.185	172±24.8	3/10
Case4							
Baseline	421±50.0	141±16.5	196±24.8	617	0.186	154±28.5	2/10
6month	466±53.6	143±28.6	N/A[B]	N/A[B]	0.21	167±39	3/10
12month	409±68.9	119±16.3	332±76.2	741	0.153	184±42.6	0/10
Case5							
Baseline	488±49.5	157±23.9	158±20.4	647	0.24	188±27.3	1/10
6month	640±35.4	224±16.5	255±11.2	895	0.449	176±19.2	5/10
12month	533±51.7	208±19.7	232±17.3	765	0.348	202±23.5	2/10

Total length of villus was calculated as the sum of villus height and crypt depth. [A]N/A: Not available due to sample loss. [B]N/A: Not available due to missing crypt depth data.
